# Regulated Intramembrane Proteolysis and Degradation of Murine Epithelial Cell Adhesion Molecule mEpCAM

**DOI:** 10.1371/journal.pone.0071836

**Published:** 2013-08-29

**Authors:** Matthias Hachmeister, Karolina D. Bobowski, Sebastian Hogl, Bastian Dislich, Akio Fukumori, Carola Eggert, Brigitte Mack, Heidi Kremling, Sannia Sarrach, Fabian Coscia, Wolfgang Zimmermann, Harald Steiner, Stefan F. Lichtenthaler, Olivier Gires

**Affiliations:** 1 Department of Otorhinolaryngology, Head and Neck Surgery, Ludwig-Maximilians-University, Munich, Germany; 2 German Center for Neurodegenerative Diseases (DZNE), Munich, Germany; 3 Technische Universität München, Munich, Germany; 4 German Center for Neurodegenerative Diseases (DZNE), Munich, Germany; 5 Tumor Immunology Laboratory, LIFE Center, Klinikum Grosshadern, Ludwig-Maximilians-University, Munich, Germany; 6 Adolf Butenandt Institute, Biochemistry, Ludwig Maximilians University, Munich, Germany; 7 Munich Center for Systems Neurology (SyNergy), Munich, Germany; INRS, Canada

## Abstract

Epithelial cell adhesion molecule EpCAM is a transmembrane glycoprotein, which is highly and frequently expressed in carcinomas and (cancer-)stem cells, and which plays an important role in the regulation of stem cell pluripotency. We show here that murine EpCAM (mEpCAM) is subject to regulated intramembrane proteolysis in various cells including embryonic stem cells and teratocarcinomas. As shown with ectopically expressed EpCAM variants, cleavages occur at α-, β-, γ-, and ε-sites to generate soluble ectodomains, soluble Aβ-like-, and intracellular fragments termed mEpEX, mEp-β, and mEpICD, respectively. Proteolytic sites in the extracellular part of mEpCAM were mapped using mass spectrometry and represent cleavages at the α- and β-sites by metalloproteases and the b-secretase BACE1, respectively. Resulting C-terminal fragments (CTF) are further processed to soluble Aβ-like fragments mEp-β and cytoplasmic mEpICD variants by the g-secretase complex. Noteworthy, cytoplasmic mEpICD fragments were subject to efficient degradation in a proteasome-dependent manner. In addition the γ-secretase complex dependent cleavage of EpCAM CTF liberates different EpICDs with different stabilities towards proteasomal degradation. Generation of CTF and EpICD fragments and the degradation of hEpICD via the proteasome were similarly demonstrated for the human EpCAM ortholog. Additional EpCAM orthologs have been unequivocally identified *in silico* in 52 species. Sequence comparisons across species disclosed highest homology of BACE1 cleavage sites and in presenilin-dependent γ-cleavage sites, whereas strongest heterogeneity was observed in metalloprotease cleavage sites. In summary, EpCAM is a highly conserved protein present in fishes, amphibians, reptiles, birds, marsupials, and placental mammals, and is subject to shedding, γ-secretase-dependent regulated intramembrane proteolysis, and proteasome-mediated degradation.

## Introduction

Regulated intramembrane proteolysis (RIP) is an important means of regulation for a growing number of transmembrane proteins [1], [2]. Through the function of various sheddases and the multiprotein γ-secretase complex comprised of minimally presenilin, APH-1, PEN-2, and nicastrin, RIP substrates are sequentially cleaved to release an extracellular ectodomain and an intracellular domain (ICD). Amyloid precursor protein (APP) is a prototype RIP substrate that was analysed in great detail owing to its central function in the pathophysiology of Alzheimer’s disease. Apart from an ectodomain and an ICD, processing of APP additionally results in the formation of a small peptides corresponding to the remainder of the extracellular domain after shedding and parts of the transmembrane domain [3]. One of these peptides, termed β-amyloid (Aβ), can misfold and form plaques in brains of Alzheimer’s disease patients. Numerous proteins have now been described to follow the scheme of RIP similarly to APP, *i.e.* sequential cleavages by α-secretases of the ADAM type, β-secretases such as BACE1 [4], and the γ-secretase complex. RIP substrates include Notch receptors [5], CD44 [6], L1 [7], [8], ERBB family members [9], and the tumour-associated antigen EpCAM [10], amongst others (for review see [1]). The biological roles of RIP are several-fold and include the generation of soluble extracellular domains with ligand activity, formation of Aβ-like peptides, release and nuclear translocation of ICDs with transcriptional capacity, and protein degradation [1], [11].

The tumour-associated antigen EpCAM (Epithelial Cell Adhesion Molecule) is a single transmembrane protein, which is highly and frequently expressed in human and rodent carcinomas, tissue progenitors, embryonic and adult stem cells [12], [13]. The first described function of EpCAM was name-giving and referred to its ability to provide epithelial cells with a weak cell adhesion through homophilic interactions of EpCAM molecules [14]. The second function of EpCAM relates to the regulation of proliferation and was in the first place described as a correlation of the expression of EpCAM with areas of proliferation in tumours [15], [16]. The involvement of EpCAM in the regulation of proliferation was in the meantime studied in more detail and reported for several conditions including tumours [17], [18], [19], [20]. Expression of EpCAM in otherwise negative cells induced the expression of the oncogene *MYC* and fostered proliferation [21]. Oppositely, the reduction of EpCAM expression correlated with diminished proliferation [17], along with a decreased expression of the cell cycle regulator cyclin D1 [22]. In patients, strong expression of EpCAM mostly correlated with a diminished overall survival [19], [23], [24], whereas some entities such as gastric cancers displayed the opposite correlation, with increased survival in the presence of high-level EpCAM expression [25]. Interestingly, even within one cancer entity, correlation of EpCAM expression with overall survival may vary across intrinsic subtypes. For example, EpCAM expression correlated with an unfavourable outcome in patients suffering from basal-like and luminal breast cancer subtypes, while high-level EpCAM expression was associated with enhanced overall survival in the HER2 subtype [26].

The mode of action of human EpCAM in the regulation of proliferation is dependent on RIP and the formation of hEpICD, the intracellular domain of EpCAM [10]. Human EpICD, comparably to Notch-ICD, translocates into the nucleus of carcinoma cells in combination with the adaptor protein FHL2 and β-catenin to bind together with LEF1 to consensus sequences within promoters of target genes including cyclin D1 [22]. Similarly to Notch-ICD and ICDs of other RIP substrates [27], [28], [29], the detection of endogeneous hEpICD was complicated and required very large amounts of protein lysate in combination with immunoprecipitation, and suggested substantial unstableness of this protein fragment [10]. Additionally to proliferation, EpCAM cleavage apparently plays a role in the maintenance of the pluripotent state of human embryonic stem cells and the regulation of differentiation, where EpICD binds to the promoters of pluripotency genes such as MYC, NANOG, POU5F1 (Oct3/4), SOX2, and KLF4 [30]. Consequently, over-expression of both, full-length EpCAM or EpICD, significantly enhanced reprogramming efficiency in mouse embryonic fibroblasts [31]. Comparably, the murine ortholog of EpCAM (mEpCAM), which shares high sequence homology with the human counterpart, was reported to be relevant to the maintenance of a pluripotent state of embryonic stem cells [32]. High-level expression of mEpCAM was also a hallmark of murine epithelial stem cells of the lung, which were additionally characterised by the expression of CD49f and CD104, low expression of CD24, and were capable of generating airway, alveolar and mixed lung epithelia [33]. Thus, mEpCAM appears to be involved in the regulation of the differentiation state in murine cells, too.

To the best of our knowledge, regulated intramembrane proteolysis of EpCAM was formally demonstrated solely for the case of the human protein so far. Here, we investigated the potential cleavage of mEpCAM and describe for the first time α-, β-, and γ-secretase proteolytic sites along with the generation of soluble EpEX, Aβ-like fragments, and different variants of mEpICD, which are highly prone to degradation by the proteasome. Comparison of EpCAM sequences across species disclosed a high homology within β- and γ-cleavage sites, and stronger heterogeneity within α- and ε-cleavage sites. We further demonstrate that human EpICD, similarly to mEpICD, is a target of proteasomal degradation. Thus, regulated intramembrane proteolysis and proteasomal degradation appear as a general theme of regulation of the function and stability of EpCAM within vertebrates.

## Results

### Murine EpCAM is Subject to Regulated Intramembrane Proteolysis

**Figure 1:**
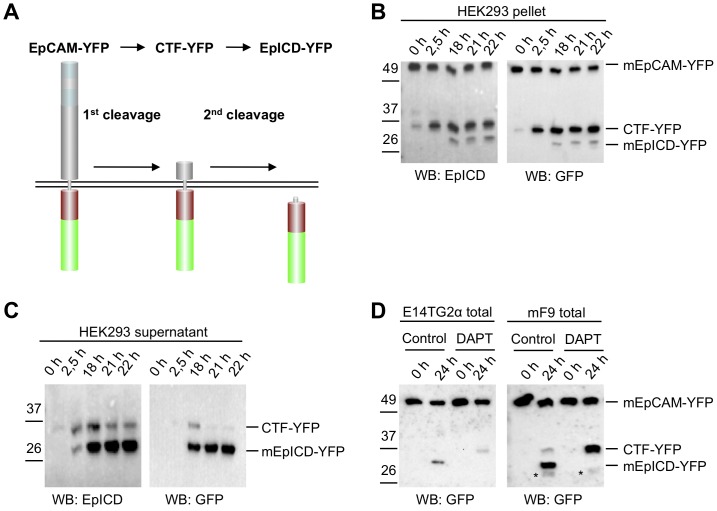
Cleavage of murine EpCAM in membrane assays. HEK293 cells were stably transfected with full-length mEpCAM in fusion with YFP (EpCAM-YFP). (**A**) Schematic representation of cleavage processes resulting in the generation of soluble EpEX, CTF-YFP, and intracellular EpICD-YFP fragments. (**B–C**) Membranes of stable transfectants were isolated and either kept at 0°C (0 h) or incubated at 37°C in reaction buffer for the indicated time points. Thereafter, pellets and supernatant were collected upon differential centrifugation. Pellets (**B**) and supernatants (**C**) of membrane assays were separated in a 10% SDS-PAGE and probed with mEpICD- and YFP-specific antibodies. (**D**) Embryonic stem cell line E14TG2a and teratocarcinoma cells mF9 were treated with DMSO (control) or the γ-secretase inhibitor DAPT before being subjected to a membrane assay. The total fraction of the membrane assay was separated in a 10% SDS-PAGE, and probed with a YFP-specific antibody. Treatment with DAPT resulted in the accumulation of CTF-YFP and in the inhibition of mEpICD-YFP formation. Protein bands corresponding to mEpCAM-YFP, CFT-YFP, and mEpICD-YFP are indicated in each immunoblot. Shown are the representative results of three independent experiments.

Regulated intramembrane proteolysis (RIP) of human EpCAM was reported in carcinoma and HEK293 cells [10]. Similarly to these previous reports, the murine ortholog of EpCAM (mEpCAM) was fused to yellow fluorescent protein (YFP) to increase the size of cleavage products and to facilitate their detection (see schematic view in Figure 1A). Cleavage and functionality of hEpCAM-YFP/hEpICD-YFP were demonstrated in earlier approaches *in vitro* and *in vivo* [10]. Cleavage was investigated in isolated membranes of stable HEK293 transfectants expressing full-length mEpCAM-YFP as described before [34]. Isolated membranes of these cells were incubated for 0 h to 22 h in a time course at 37°C to allow for cleavage to occur. Thereafter, membranous and soluble fractions were harvested separately via differential centrifugation and the presence of cleaved variants of mEpCAM determined in immunoblot experiments with mEpICD- and YFP-specific antibodies. Three distinct proteins were detected with mEpICD- and YFP-specific antibodies in particulate fractions of membrane-based assays (Figure 1B). Apparent molecular masses were calculated using the Chemidoc XRS+ imaging system and corresponded to the predicted molecular mass for mEpCAM-YFP (predicted: 62.55 kDa; apparent: 66.7 kDa), CTF-YFP (predicted: 34.5–37 kDa; apparent: 34.9 kDa), and mEpICD-YFP (predicted: 31 kDa; apparent: 29.9 kDa). These molecular weights refer to fusions with YFP, hence 25 kDa must be subtracted to determine actual EpCAM fragment sizes. Only small amounts of the C-terminal fragment mCTF-YFP were present at the initial time point, which might reflect the overall status of mEpCAM cleavage at the time of membrane isolation. At this time point, a major mCTF fragment with an approximate molecular weight of 34.9 kDa represented the dominant mCTF band. Two additional bands of weaker intensity and with molecular weights of 37 kDa and 40 kDa were detected using mEpICD-specific antibodies (Figure 1B). Upon time, these two proteins disappeared and after 2.5 h, the level of the 34.9 kDa mCTF-YFP strongly increased and remained stable over the observation time of 22 h (Figure 1B). At later time points, we observed the appearance of a smaller mEpICD-reactive protein, which corresponded to mEpICD-YFP, in comparably small amounts (Figure 1B). All three major protein species were identified with mEpICD and YFP-specific antibodies. In supernatants of membrane assays, two mEpICD and YFP-reactive proteins were detected, which corresponded to the 34.9 kDa mCTF-YFP and mEpICD-YFP (Figure 1C). The ratio of mCTF-YFP to mEpICD-YFP was inversed in supernatants versus pellets, in line with the notion that mEpICD is release from membrane-bound mCTF-YFP as a soluble protein. Residual levels of mEpCAM-YFP and mCTF-YFP occasionally seen in supernatants and of mEpICD-YFP in pellets might represent minor cross-contaminations of subcellular fractions.

Next, we analysed the potential cleavage of mEpCAM-YFP in murine embryonic stem cells (ESCs; E14TG2α) and in teratocarcinoma cells (mF9). Stable transfectants were subjected to membrane assays and resulting YFP-reactive proteins were detected in immunoblot experiments at late time points to ensure quantitative cleavage. In both cell lines, generation of mEpICD-YFP was observed after 24 h and was most prominent in mF9 cells (Figure 1D). Additionally, mCTF-YFP was detected in mF9 cells along with a protein band corresponding to YFP only, probably due to degradation (labelled * in Figure 1D). Inhibition of the γ-secretase complex, which was reported to be involved in cleavage of human EpCAM [10], with the synthetic inhibitor DAPT resulted in the accumulation of mCTF-YFP and the lack of detectable mEpICD in both cell lines (Figure 1D). Thus, mEpCAM is subject to γ-secretase-dependent RIP.

### Determination of Cleavage Sites of Sheddases within EpCAM

**Figure 2:**
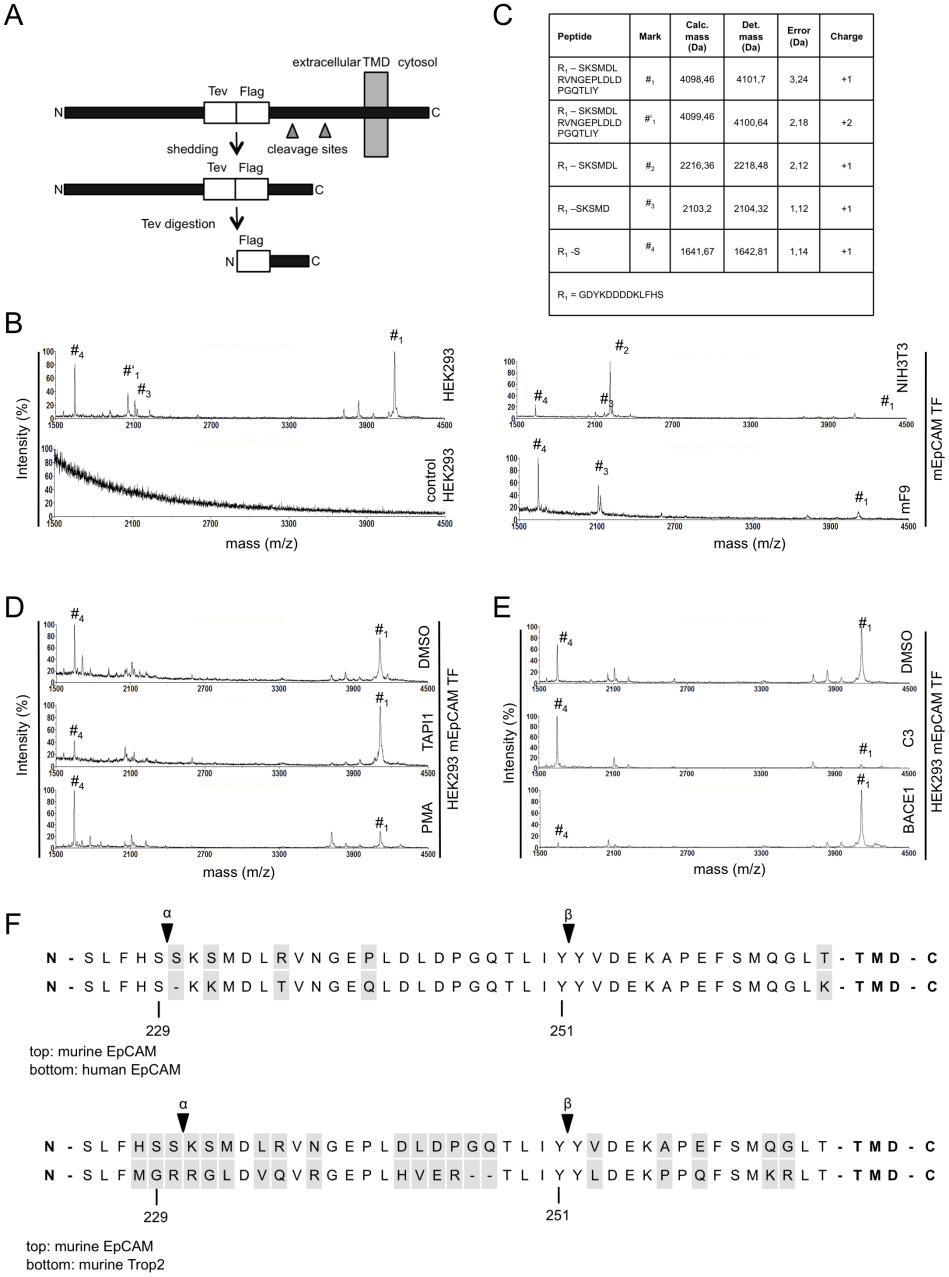
Determination of the sheddase cleavage amino acid sequence in mEpEX. (**A**) Schematic representation of mEpCAM-TF containing a TEV protease recognition site and a Flag-Tag in the mEpEX domain 42 amino acids before the predicted transmembrane domain. After cleavage by sheddases, the largest part of mEpEX can be removed through digestion with TEV protease and the resulting small fragment immunoprecipitated using Flag-specific antibodies. (**B**) Representative mass spectrometry spectrum of HEK293, NIH3T3, and mF9 cells stably expressing mEpCAM-TF and of vector control HEK293 cells as a control. Four major peak species are indicated. (**C**) Tabular overview of sheddase cleavage sites within mEpEX as determined upon mass spectrometric analysis and alignment to potential molecular weights. Calculated and determined masses are given in Dalton including error and charge of each peptide. (**D**) Representative mass spectrometry spectrum of HEK293 cells stably expressing mEpCAM-TF after treatment with DMSO, the metalloprotease protease inhibitor TAPI-1, and the phorbol ester PMA. (**E**) Representative mass spectrometry spectrum of HEK293 cells stably expressing mEpCAM-TF after treatment with DMSO, the BACE1 protease inhibitor C3, and after transient transfection of BACE1 expression plasmid. (**F**) Sequence alignment of murine and human EpCAM (top), and murine EpCAM and murine Trop-2 (bottom). metalloprotease protease cleavage (a-secretase) and BACE1 cleavage sites (b-secretase) are indicated.

In the following, we intended to identify cleavage sites within mEpCAM at the amino acid level using mass spectrometry approaches. RIP is a sequential process, which involves the initial cleavage of proteins by sheddase(s) in the extracellular domain and intramembrane proteases. The extracellular domain of mEpCAM is strongly glycosylated, which would hinder proper analysis of the resulting peptides in mass spectrometry. For this reason, similarly to a previous study [35] two peptide motifs (tags) were incorporated 42 amino acids away from the predicted transmembrane domain within the extracellular domain of mEpCAM in order to generate mEpCAM-TF. Incorporation of these tags did not impair the cleavage of substrates such as amyloid precursor-like protein 2 (APLP2) and were therefore used in the present study [35]. The first motif represents a consensus site for the tobacco etch virus (TEV) protease, while the second encodes the FLAG tag. After cleavage, the resulting mEpEX protein can be immunoprecipitated from cell culture supernatants with FLAG-specific antibodies and the greatest part of the protein removed upon digestion with TEV protease (Figure 2A). The remaining fragment is small and directly amenable to mass spectrometric analysis. mEpCAM-TF was stably transfected in HEK293 cells, mF9 cells, and murine NIH3T3 fibroblasts. Cell culture supernatants of each stable transfectant were subjected to FLAG immunoprecipitation, TEV digestion, and subsequent mass spectrometric analysis of immunoprecipitates. The resulting spectra displayed a total of four prominent peaks (Figure 2B) with determined molecular masses of 4101.70 Da (peak #1), 2218.48 Da (peak #2), 2104.32 Da (peak #3), and 1642.81 Da (peak #4) (Figure 2C). These four peaks were absent in control HEK293 cells stably transfected with the pCAG expression vector and in wild-type, untransfected HEK293 cells (Figure 2B, lower left panel and data not shown). Each peptide was assigned to calculated molecular masses and thereby identified at the single amino acid level (Figure 2C). All four peptides carried a single positive charge with the exception of peak #1, which also occurred as a variant with a double positive charge (peak #′1). The double charge of this fragment was confirmed in measurements performed in reflector mode upon the mass differences in the isotope pattern. Accordingly, peak #′1 displayed a halved mass-over-charge ratio in the mass spectrometry spectra (Figure 2B). All other masses were additionally recorded in reflector mode, confirming the data and yielding masses with high accuracy and small variation to calculated molecular masses (data not shown). Using MALDI-ToF devices in reflector mode allows for an increased time of flight of the ion of interest and, hence, for an improved resolution of spectra and mass accuracy.

Prototype RIP substrates can be cleaved by a-secretases, typically metalloproteases of the ADAM (A Disintegrin And Metalloprotease) family and by β-secretase such as BACE1 (Beta-site APP Cleaving Enzyme 1). In order to assign peptide peaks to potential proteases, HEK293 cells expressing mEpCAM-TF were treated with the broadband metalloprotease inhibitor TAPI-1. In comparison to control-treated cells (DMSO), intensity of peak #4 was decreased by 60% upon TAPI-1 treatment relative to peak #1 (Figure 2D). In contrast, peak #4 became the dominant peak in spectra of cells treated with the phorbol ester PMA (Figure 2D), which enhances the activity of ADAM proteases [36]. In parallel, HEK293 cells expressing mEpCAM-TF were treated with the BACE1 inhibitor C3 and subjected to mass spectrometric analysis. Inhibition of BACE1 induced a reduction of peak #1 by more than 90% relative to peak #4, while transient expression of BACE1 resulted in a strong increase of peak #1 to become the dominant peak (Figure 2E). These findings were further substantiated by mass spectrometry measurements in reflector mode. Thus, mEpCAM is subject to extracellular cleavage by a-secretases and is a novel substrate of the β-secretase BACE1. Figure 2F displays a schematic view of the α- and β-secretase cleavage sites in mEpCAM aligned to corresponding sequences of human EpCAM. The β-secretase cleavage sequence between tyrosine^251^ and tyrosine^252^ was perfectly conserved amongst both proteins, while the α-secretase sequence between serine^230^ and lysine^231^ appeared to be displaced by one amino acid in the human sequence (Figure 2F, upper panels). Human and murine EpCAM are members of the Trop family of proteins comprised of two specimens, *i.e.* Trop-1 ( = EpCAM) and Trop-2, which was recently described to be target of RIP [37]. The β-secretase cleavage sequence was conserved in both murine family members, while the α-secretase sequence was not conserved, with a change of serine^230^ to an arginine in murine Trop-2 (Figure 2F, lower panels). A comparative alignment of EpCAM sequences with cleavage sites in reported ADAM and BACE1 substrates did not disclose any obvious consensus sequence for cleavage (Table S1).

### Determination of Cleavage Sites of g-secretase within EpCAM

**Figure 3:**
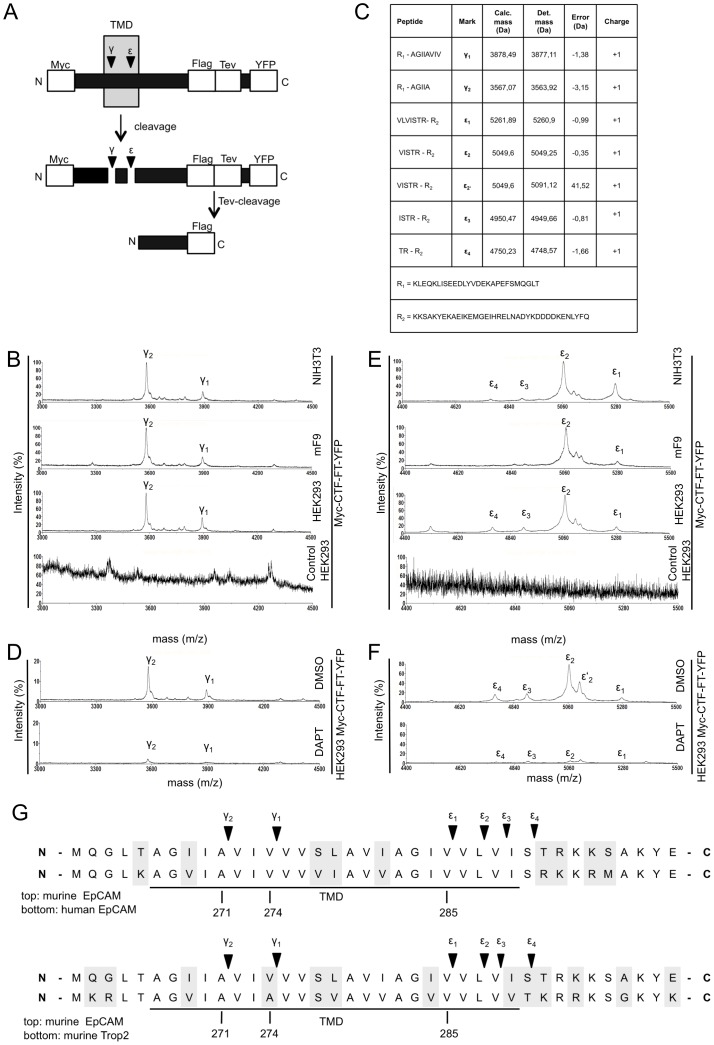
Determination of the γ-secretase cleavage amino acid sequence in mEpCAM. (**A**) Schematic representation of Myc-CTF-YFP containing an N-terminal c-Myc-tag, a Flag-Tag and a TEV protease recognition site as well as YFP C-terminally of mEpICD. After cleavage by g-secretase, the Ab-like fragment can be isolated upon immunoprecipitation with c-Myc-specific antibodies. The YFP moiety is removed through digestion with TEV protease and the resulting small peptide isolated upon immunoprecipitation with Flag-specific antibodies. (**B**) Representative mass spectrometry spectrum of HEK293, NIH3T3, and mF9 cells stably expressing Myc-CTF-TF-YFP after immunoprecipitation of supernatants with c-Myc-specific antibodies. Two major peak species representing g-cleavages are indicated. (**C**) Tabular overview of γ-secretase cleavage sites within mEpCAM as determined upon mass spectrometric analysis and alignment to potential molecular weights. Calculated and determined masses are given in Dalton including error of each peptide. (**D**) Representative mass spectrometry spectrum of HEK293 cells stably expressing mEpCAM-TF after immunoprecipitation with c-Myc-specific antibodies and treatment with DMSO and the γ-secretase inhibitor DAPT. (**E**) Representative mass spectrometry spectrum of HEK293, mF9, and NIH3T3 stably expressing Myc-CTF-TF-YFP after TEV digestion and immunoprecipitation with Flag-specific antibodies. Four major peaks representing ε-cleavages are indicated. (**F**) Representative mass spectrometry spectrum of HEK293 cells stably expressing mEpCAM-TF after TEV digestion, immunoprecipitation with Flag-specific antibodies, and treatment with DMSO and the γ-secretase inhibitor DAPT. (**G**) Sequence alignment of murine and human EpCAM (top), and murine EpCAM and murine Trop-2 (bottom). γ-Secretase cleavages at g-position and e-position are indicated.

The above mentioned cleavage of mEpCAM by ADAM protease(s) and BACE1 results in the generation of C-terminal fragments termed CTF as seen in membrane assays. As shown above, cleavage of CTF-YFP to mEpICD-YFP was sensitive towards the treatment of cells with γ-secretase inhibitor DAPT (see Figure 1C). In order to define the cleavage sequences of γ-secretase within mEpCAM at the single amino acid level, a truncated variant of mEpCAM, which mimics mCTF-YFP, was generated. Myc-CTF-FT-YFP is comprised of an N-terminal signal sequence, an N-terminal Myc tag, 15 amino acids of the extracellular domain, and the transmembrane and intracellular domain of mEpCAM fused to YFP. Additionally, a FLAG tag and a TEV recognition site were incorporated C-terminally of the intracellular domain followed by a short linker region of 6 amino acids and the YFP moiety (Figure 3A). γ-cleavage of Myc-CTF-FT-YFP would release an Aβ-like fragment, which can be immunoprecipitated from the supernatant with Myc tag-specific antibodies. ε-cleavage of Myc-CTF-FT-YFP would release mEpICD-TF-YFP, which can be further shortened through TEV digestion and thereafter immunoprecipitated with FLAG-specific antibodies (Figure 3A). Myc-CTF-FT-YFP was stably expressed in HEK293 cells, mF9 cells, and NIH3T3 fibroblasts, and cell supernatants were used for Myc tag-specific immunoprecipitations and mass spectrometric analysis. In all three cell lines, two major peaks were detected in mass spectrometric spectra, which were not detectable in HEK293 cells stably transfected with the pCAG expression vector only of in wild-type untransfected HEK293 cells (Figure 3B and data not shown). Peak γ1 represented an Ab-like peptide of EpCAM after cleavage between amino acids valine^274^ and valine^275^, with a calculated molecular mass of 3878.49 Da and a detected molecular mass of 3877.11 Da (Figure 3C). Peak γ2 represented an Ab-like peptide of EpCAM after cleavage between amino acids alanine^271^ and valine^272^, with a calculated molecular mass of 3567.07 Da and a detected molecular mass of 3563.92 Da (Figure 3C). Treatment of HEK293 Myc-CTF-FT-YFP cells with the g-secretase inhibitor DAPT led to a >80% reduction of the intensity of both peaks (Figure 3D). Note that in Figure 3D and 3F, the “base peak relative display” function was used in the Data Explorer software instead of the standard “display relative” mode, in which peaks are set to 100% and which therefore would preclude a direct comparison of untreated and treated samples.

In parallel, lysates of stable transfectants of HEK293, mF9, and NIH3T3 cells were subjected to FLAG immunoprecipitation, TEV digestion, and elution before mass spectrometric analysis. Four major peaks were reproducibly detected in spectra of lysates, with peak ε2 representing the major peak. These four peaks were not detectable in HEK293 cells stably transfected with the pCAG expression vector only of in wild-type untransfected HEK293 cells (Figure 3E and data not shown). The first three peaks were aligned to EpICD molecules generated upon cleavage of EpCAM C-terminally of valine^285^, leucine^287^, and valine^288^ within the predicted transmembrane domain (see molecular masses in Figure 3C; Figure 3G). The fourth peak corresponded to a cleavage after serine^290^ within the predicted intracellular domain. Therefore, we assumed that this cleavage is the result of N-terminal trimming by intracellular protease(s). The mass difference of approximately 41,52 Da of peak ε2′could result from acetylation (+42 Da) or trimethylation (+42 Da) of lysine residues of the peptide stretch. Alternatively, this shift could be caused by addition of a potassium ion (+38 Da). γ-Secretase-dependent generation of the observed peptide peaks was assessed following treatment of HEK293 transfectants with DAPT. The intensity of all four major peaks was reduced by >80% (Figure 3F). Alignment of the corresponding amino acid sequences of murine and human EpCAM disclosed 100% homology at every γ-secretase cleavage site (γ- and ε-sites) defined in murine EpCAM (Figure 3G). Cleavage site γ2 and ε1–3 were also conserved between murine EpCAM and murine Trop-2, while cleavage site γ1 was changed from valine to alanine and cleavage site ε4 from serine^290^ to threonine (Figure 3G).

### EpICD is Prone to Degradation by the Proteasome

**Figure 4:**
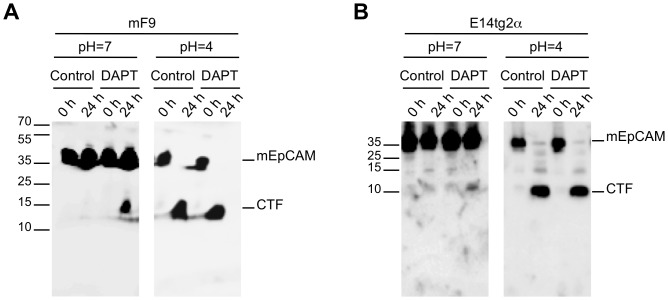
Cleavage of endogeneous mEpCAM. Proteolytic cleavage of mEpCAM was addressed in mF9 (**A**) and E14TG2a (**B**) cells using membrane assays at pH 7 and pH 4. Membranes of mF9 and E14TG2a cells were incubated for 0 h and 24 h at 37°C and EpCAM fragments were detected in immunoblots using a mEpICD-specific antibody in combination with an HRP-conjugated secondary antibody. Inhibition of the γ-secretase complex was achieved upon treatment with DAPT where indicated. Shown are the representative results of three independent experiments.

Cleavage of endogeneous mEpCAM was addressed in mF9 and E14TG2a cell lines with membrane assays. Since metalloproteases and BACE1 differ in their pH optimum, membrane assays were performed at pH7 and pH4. At pH7 and in the absence of any inhibitor, no mEpCAM fragment was detectable except for full-length mEpCAM. Inhibition of the γ-secretase complex with DAPT resulted in the accumulation of endogeneous mCTF after 24 h in teratocarcinoma (mF9) and ES cells (E14TG2α) (Figure 4A and B, left panels). Owing to the abovementioned shedding of mEpCAM by BACE1, the same experiments were performed at pH = 4, which represents the pH optimum of BACE1 [38]. In this acidic environment, endogeneous mEpCAM was quantitatively cleaved to generate mCTF, while mEpICD was not detectable possibly due to the inappropriate pH for the enzymatic activity of γ-secretase and owing to the very small size of the resulting protein (Figure 4A and B, right panels). Hence, endogeneous mEpCAM is subject to proteolytic cleavage but detection of mEpICD was technically not feasible under the assay conditions. Detection of ICDs generated through presenilin-dependent RIP is usually a very difficult task due to rapid and efficient degradation of ICDs after release into the intracellular space [27], [28], [29]. Detection of human EpICD was highly inefficient and achieved only upon immunoprecipitation of large amounts of protein lysate in the milligram range. This led the authors to the notion that hEpICD is a very small protein, whose biochemical properties hamper thorough detection and to the assumption of a potential degradation of hEpICD through the proteasome [10].

**Figure 5:**
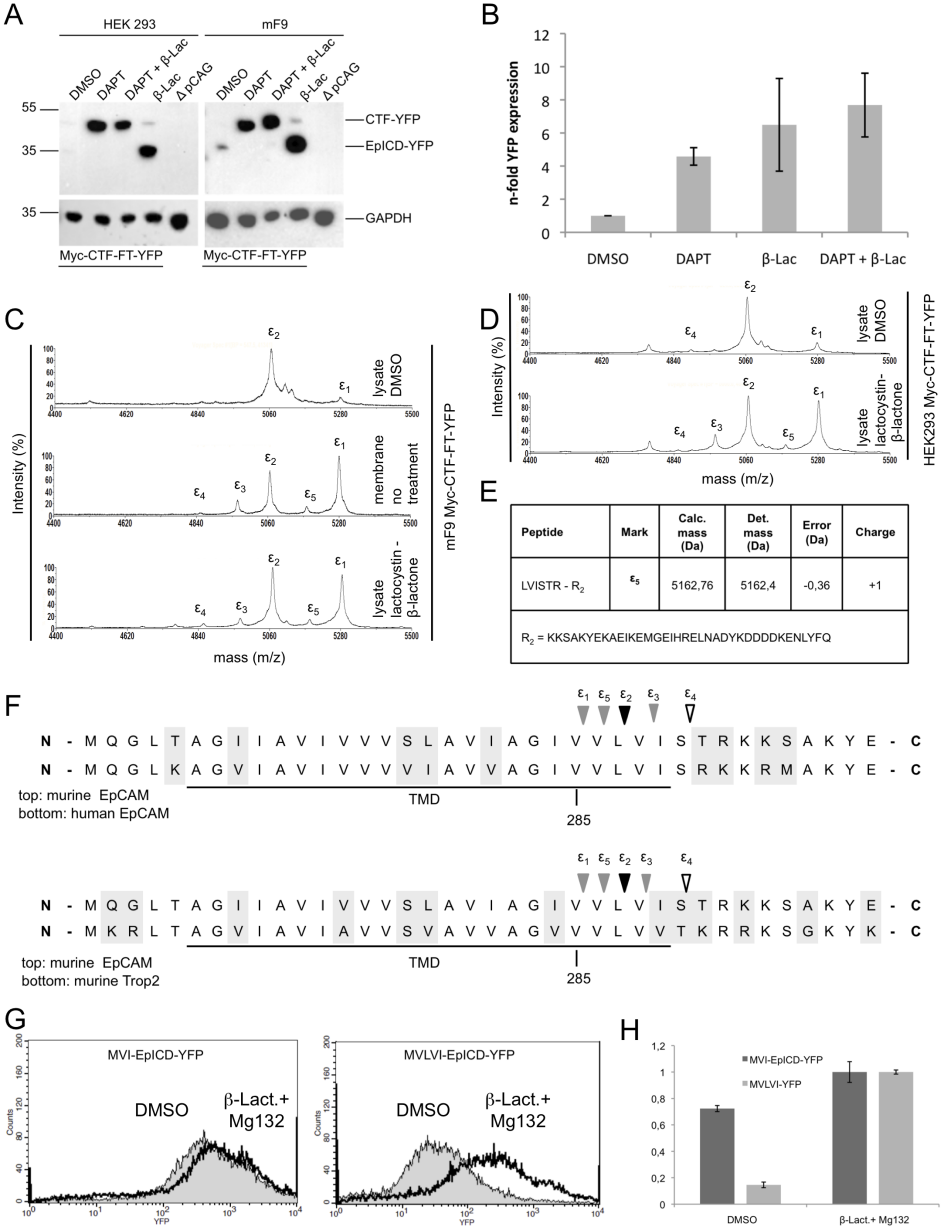
Stability of mEpICD species. (**A**) HEK293 and mF9 cells stably expressing murine Myc-CTF-FT-YFP were treated with DMSO, DAPT, lactacystin-β-lactone, or DAPT and lactacystin-β-lactone. Protein lysates were separated in a 10% SDS-PAGE and probed with a YFP-specific antibody. As a control, lysates from vector control HEK293 cells were used (WT). CTF-YFP and EpICD-YFP are annotated. As a loading control, the same immunoblot membranes were probed with GAPDH-specific antibodies. (**B**) mF9 cells stably expressing murine Myc-CTF-FT-YFP were treated with DMSO, DAPT, lactacystin-β-lactone, or DAPT and lactacystin-β-lactone. YFP fluorescence intensities were assessed upon flow cytometry and normalised to values of DMSO-treated controls. Shown are mean values with standard deviations from three independent experiments. (**C**) mF9 cells stably expressing murine Myc-CTF-FT-YFP were subjected to mass spectrometric analysis. To do so, lysates of cells treated with DMSO or the proteasome inhibitor lactacystin-β-lactone and lysates from membrane assays were used. Representative mass spectrometry spectrum is depicted and five major peaks are annotated. Membrane assay probes and treatment with proteasome inhibitor led to the increase of peak ε1 and to the appearance of two peaks ε3 and ε5. (**D**) HEK293 cells stably expressing murine Myc-CTF-FT-YFP were treated with DMSO or the proteasome inhibitor lactacystin-b-lactone. Representative mass spectrometry spectrum is depicted and five major peaks are annotated. (**E**) Tabular overview of γ-secretase cleavage sites within mEpCAM as determined upon mass spectrometric analysis and alignment to potential molecular weights. Calculated and determined masses are given in Dalton including error of the peptide. (**F**) Sequence alignment of murine and human EpCAM (top), and murine EpCAM and murine Trop-2 (bottom). γ-Secretase cleavages at ε-position are indicated. Solid triangle marks the cleavage site of the stable EpICD variant, grey triangles cleavage sites of labile EpICD variants, and open triangle of N-terminally trimmed EpICD. (**G**) HEK293 cells stably expressing murine MVI-EpICD-YFP and MVLVI-EpICD-YFP mutants were subjected to flow cytometry assessment of YFP fluorescence. Shown are representative graphs of both stable transfectants after treatment with DMSO or lactacystin-β-lactone. (**H**) YFP fluorescence intensities of HEK293 transfectants stably expressing murine MVI-EpICD-YFP and MVLVI-EpICD-YFP mutants are given as mean fluorescence intensity ratios. Cells treated with lactacystin-β-lactone served as reference and values were set to one for comparison.

Therefore, we addressed the cleavage and stability of mEpICD using the mCTF-FT-YFP construct, which is readily processed by γ-secretase, in stable transfectants of HEK293 cells. In line with an anticipated poor stability of mEpICD, mCTF-YFP was very weakly and mEpICD-YFP was not detectable in immunoblot experiments with whole cell lysates of HEK293 and mF9 cells stably expressing Myc-CTF-FT-YFP in the absence of any treatment (Figure 5A, DMSO lane). Treatment of cells with the proteasome inhibitor lactacystin-β-lacton or MG132 strongly stabilised mEpICD and allowed for the detection of substantial amounts of cleaved mEpICD (Figure 5A, lanes 4 and data not shown). Further experiments were conducted with lactacystin-β-lacton because MG132 was reported to be a pleiotropic drug, which affects the enzymatic activity of β- and β-secretase to substantial degree, too [39], [40], [41]. Interestingly, treatment of cells with the β-secretase inhibitor DAPT resulted in strong stabilisation and accumulation of Myc-CTF-FT-YFP, suggesting that primarily mEpICD and not mCTF is prone to proteasomal degradation (Figure 5A, lanes 2 and 3). Accordingly, treatment of cells with lactacystin-β-lacton induced only a minor stabilisation of Myc-CTF-FT-YFP (Figure 5A, lanes 4). The specificity of all protein bands was confirmed using lysates from HEK293 cells transfected with the empty vector only (Figure 5A).

YFP fluorescence was used as a surrogate marker in flow cytometry experiments for the quantification of Myc-CTF-FT-YFP derivatives after DAPT and lactacystin-β-lacton treatment. YFP fluorescence of control-treated cells (DMSO) was set to one for a comparison. Treatment of cells with the g-secretase inhibitor DAPT, the proteasome inhibitor lactacystin-β-lacton, or a combination of both resulted in 4.5-fold, 6.5-fold, and 7.7-fold increase in YFP fluorescence compared to DMSO, respectively (Figure 5B). Hence, inhibition of cleavage of Myc-CTF-FT-YFP to mEpICD-YFP or inhibition of proteasomal activity stabilised YFP fluorescence to a comparable degree. We concluded from these results that cleavage of Myc-CTF-FT-YFP to generate mEpICD-YFP is required for proteasome-dependent degradation.

Mass spectrometric analysis of EpICD disclosed the existence of variants, which differed in their N-terminal amino acids and in peak intensities, suggesting differential generation and/or degradation rates (see Figure 3). In order to address a possible differential degradation of these mEpICD variants, mEpICD variants from mF9 cells expressing Myc-CTF-FT-YFP were retrieved from cell lysates or from membrane assays (*i.e.* in the absence of cytosolic proteases). mF9 cells were chosen because of the strong cleavage capacity observed in immunoblot experiments (See Figure 1C). Mass spectrometric analysis of mEpICD confirmed the presence of one major variant in mF9 cells (Figure 5C, upper spectrum, peak e2). Additionally, a second minor peak termed ε1 was detected (Figure 5C). In contrast, five variants of mEpICD were generated in membrane assays, with peak ε3–5 representing novel components and a substantial increase in the intensity of peak ε1, which became the dominant peak (Figure 5C, middle panel). A comparable spectrum was obtained with whole cell lysates of mF9 cells treated with the proteasome inhibitor lactacystin-b-lacton, confirming a selective proteasome-mediated degradation of mEpICD variants (Figure 5C, lower panel). Effects of the inhibition of the proteasome were recapitulated in HEK293 cells stably expressing Myc-CTF-FT-YFP with a resulting increase of peak ε1 and the generation of peak ε3 and ε5 after lactacystin-b-lacton treatment (Figure 5D). The amino acid sequence of peak ε5 was aligned to calculated molecular mass (Figure 5E) and represents an mEpICD variant generated upon cleavage after valine^286^ (Figure 5F). Comparisons of the amino acid sequences of murine and human EpCAM and of murine EpCAM and murine Trop-2 in the region of ε-cleavage are shown in Figure 5F. Amino acid sequences were identical in murine and human EpCAM, while cleavage position ε4 was exchanged from serine to threonine between mEpCAM and Trop-2 (Figure 5F). Hence, e-cleavage of mEpCAM generates mEpICD variants, which differ in their N-terminal amino acid composition and in their sensitivity towards proteasome-mediated degradation within the cell. Peak ε2 corresponded to a mEpICD variant starting with valine^288^-isoleucine^289^ and represented the most stable mEpICD variant. In order to substantiate these findings, two versions of soluble mEpICD were cloned, which contained the N-terminal motifs methionine-valine-leucine-valine-isoleucine (MVLVI; mimic of peak ε1) and methionine-valine-isoleucine (MVI; mimic of peak ε2). MVLVI-EpICD-YFP and MVI-EpICD-YFP were stably introduced into HEK293 cells and their respective expression was monitored by flow cytometric measurement of YFP fluorescence. Treatment of stable transfectants with the proteasome inhibitors lactacystin-b-lacton and MG132 had only a minor impact on the expression of the MVI-EpICD-YFP variant, while it strongly increased the expression of MVLVI-EpICD-YFP (Figure 5G). Quantification of the effects of inhibitors of the proteasome on the stability of both mEpICD variants was conducted with YFP fluorescence values, where fluorescence of treated cells was set to one. In the absence of inhibitors, the expression of MVI-EpICD was decreased to 70% of inhibitor-treated cells, while the expression of MVLVI-EpICD was reduced to 15% (Figure 5H). Thus, the extended variant of mEpICD, which included an additional valine and a leucine, was significantly more prone to proteasomal degradation. According to the N-end rule, differing N-terminal amino acids regulate the stability of proteins. However, both EpICD variants display an N-terminal valine residue and, furthermore, mutation of either leucine or isoleucine in the second position did not alter the stability of the resulting EpICD variants (*data not shown*).

### Human EpICD is Prone to Proteasome-dependent Degradation

**Figure 6:**
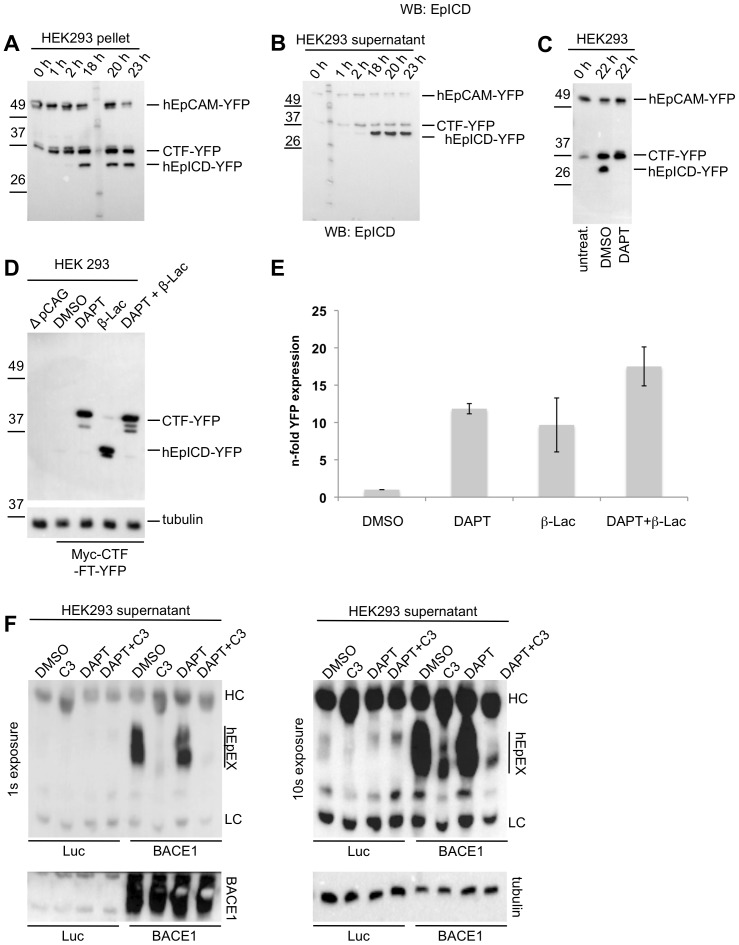
Cleavage and proteasomal degradation of human EpCAM. HEK293 cells were stably transfected with hEpCAM-YFP and used to determine cleavage products of hEpCAM in membrane assays. Membranes of stable transfectants were isolated and either kept at 0°C (0 h) or incubated at 37°C in reaction buffer for the indicated time points. Thereafter, pellets and supernatant were collected upon differential centrifugation. Pellets (**A**) and supernatants (**B**) of membrane assays were separated in a 10% SDS-PAGE and probed with hEpICD- and YFP-specific antibodies. (**C**) HEK293 hEpCAM-YFP transfectants were treated with DMSO (control) or the γ-secretase inhibitor DAPT before being subjected to a membrane assay. The total fraction of the membrane assay was separated in a 10% SDS-PAGE, and probed with a YFP-specific antibody. Treatment with DAPT resulted in the accumulation of CTF-YFP and in the inhibition of hEpICD-YFP formation. (**D**) HEK293 human Myc-CTF-TF-YFP transfectants were treated with DMSO (control), the γ-secretase inhibitor DAPT, the proteasome inhibitor β-lacto-lactocystin (β-Lac), or combination of both. Thereafter, whole cell lysates were separated in a 10% SDS-PAGE, and probed with a YFP-specific antibody. Treatment with β-lacto-lactocystin resulted in an accumulation of hEpICD-YFP. Similar loading of protein lysates was visualised upon staining of tubulin on the same blots. Protein bands corresponding to human Myc-CTF-TF-YFP and mEpICD-YFP are indicated in each immunoblot. Shown are the representative results of three independent experiments. (**E**) YFP fluorescence was analysed in dependency of the treatment of HEK293 human Myc-CTF-TF-YFP transfectants. DMSO treatment served as a reference and values were normalised to one. Shown are the mean values with standard deviations from three independent experiments. (**F**) HEK293 cells stably expressing hEpCAM-YFP were transiently transfected with expression plasmids for luciferase (Luc) as a control or BACE1 (BACE1). After 24 hours, supernatants were removed and cells treated with the indicated inhibitors of BACE1 (C3), γ-secretase (DAPT) or combinations thereof. After additional 24 hours, supernatants were collected and hEpEX was immunoprecipitated and visualised upon immunoblotting with specific antibodies. Shown are two representative results with exposure times (1 s and 10 s). Over-expression of BACE1 and equal protein loading were verified upon immunobloting (lower left and right panel, respectively).

In order to specify the cleavage of human EpCAM, membrane assays were performed with HEK293 cells stably expressing hEpCAM-YFP. Over time, an accumulation of two C-terminal fragments of human EpCAM, which preceded the generation of hEpICD-YFP, was observed (Figure 6A and B). Similarly to mEpCAM-YFP, the amount of CTF-YFP and hEpICD-YFP in pellets and soluble fractions of membrane assays were reciprocal, with hEpICD amounts being highest in the soluble fraction (Figure 6B). Treatment of stable transfectants of HEK293 cells with the γ-secretase inhibitor DAPT resulted in a loss of hEpICD, confirming the involvement of γ-secretase in the cleavage of CTF-YFP to hEpICD-YFP (Figure 6C).

In a next step, the stability of cleavage products of CTF-YFP was addressed in whole cell lysates of HEK293 transfectants and thus in the presence of intracellular proteases. Barely visible amounts of hEpICD-YFP were detected in DMSO-treated transfectants, whereas treatment with DAPT stabilised CTF-YFP (Figure 6D). Interestingly, treatment of transfectants with the proteasome inhibitor lactacystin-b-lacton allowed for the visualisation of substantial amounts of hEpICD-YFP (Figure 6D). The fluorescence of YFP was used as a surrogate marker to measure the expression of CTF-YFP and hEpICD-YFP in dependency of the treatment of cells with γ-secretase and/or proteasome inhibitor. Inhibition of γ-secretase or the proteasone resulted in a >10-fold and 10-fold increase in YFP fluorescence, respectively (Figure 6E). Treatment of cells with a combination of both inhibitors did not result in an additive effect, suggesting that proteasomal degradation is a major pathway of hEpICD-YFP destruction (Figure 6E).

Initial reports on the cleavage of human EpCAM primarily described ADAM17 as sheddase. The finding of BACE1 as a novel protease involved in cleavage of the extracellular domain of mEpCAM prompted us to study the cleavage of hEpCAM by BACE1. HEK293 cells stably expressing hEpCAM-YFP were transiently transfected with either a luciferase or a BACE1 expression plasmid. After 24 hrs, cells were treated with DMSO, the BACE1 inhibitor C3, DAPT or a combination of C3 and DAPT. After additional 24 hrs, supernatants were collected and hEpEX was immunoprecipitated and visualised in immunobloting using specific antibodies. Expression of BACE1 induced a strong cleavage of hEpCAM-YFP, which was substantially inhibited upon treatment with C3 but not DAPT (Figure 6F). As seen in longer exposures, EpEX shedding in the presence of high-level BACE1 were far superior to those in luciferase-transfected cells (Figure 6F, lower panel). Levels of BACE1 expression were controlled in whole cell lysates after transient transfection and disclosed very high levels of BACE1 (Figure 6F, lower left panel). Hence, human EpCAM is also a substrate for the BACE1 secretase.

### EpCAM is a Conserved Protein Present throughout Vertebrates

EpCAM was mainly described and characterised in human [10], mouse [32], rat [42], and zebrafish cells [43]. Here, we describe the regulated intramembrane proteolysis of the murine ortholog of EpCAM, which was strongly reminiscent of cleavage of human EpCAM. We searched for EpCAM orthologs *in silico* with the amino acid sequence of human EpCAM as a reference (NP_002345.2) with the aim to study the conservation of EpCAM and its cleavage sequences. Combination of blastp and UniProt searches allowed for the retrieval of 61 partial or complete amino acid sequences, which could represent putative EpCAM orthologs in fishes, amphibians, reptiles, birds, a monotreme, marsupials, ungulates, primates, and rodents. Species’ names, trivial names, accession numbers, amino acid sequence identity to the human EpCAM sequence, amino acid conservation, predicted amino acid counts, and gaps compared to human EpCAM are given in Table S2 and Figure S1. EpCAM orthologs were not found in plants, bacteria, viruses, and fungi using available databases. Lowest sequence identities were found between human EpCAM and orthologs in fishes (39–46%), while highest sequence identities were found with orthologs in primates (90–99%) (Table S2). Most primates contained predicted EpCAM orthologs with amino acid numbers identical to human EpCAM (314 amino acids), whereas shorter variants of EpCAM were observed commonly in fishes. Zebrafish EpCAM is comprised of 302 amino acids and lacks 7 amino acids from the leader peptide and the 5 most C-terminal amino acids.

**Figure 7:**
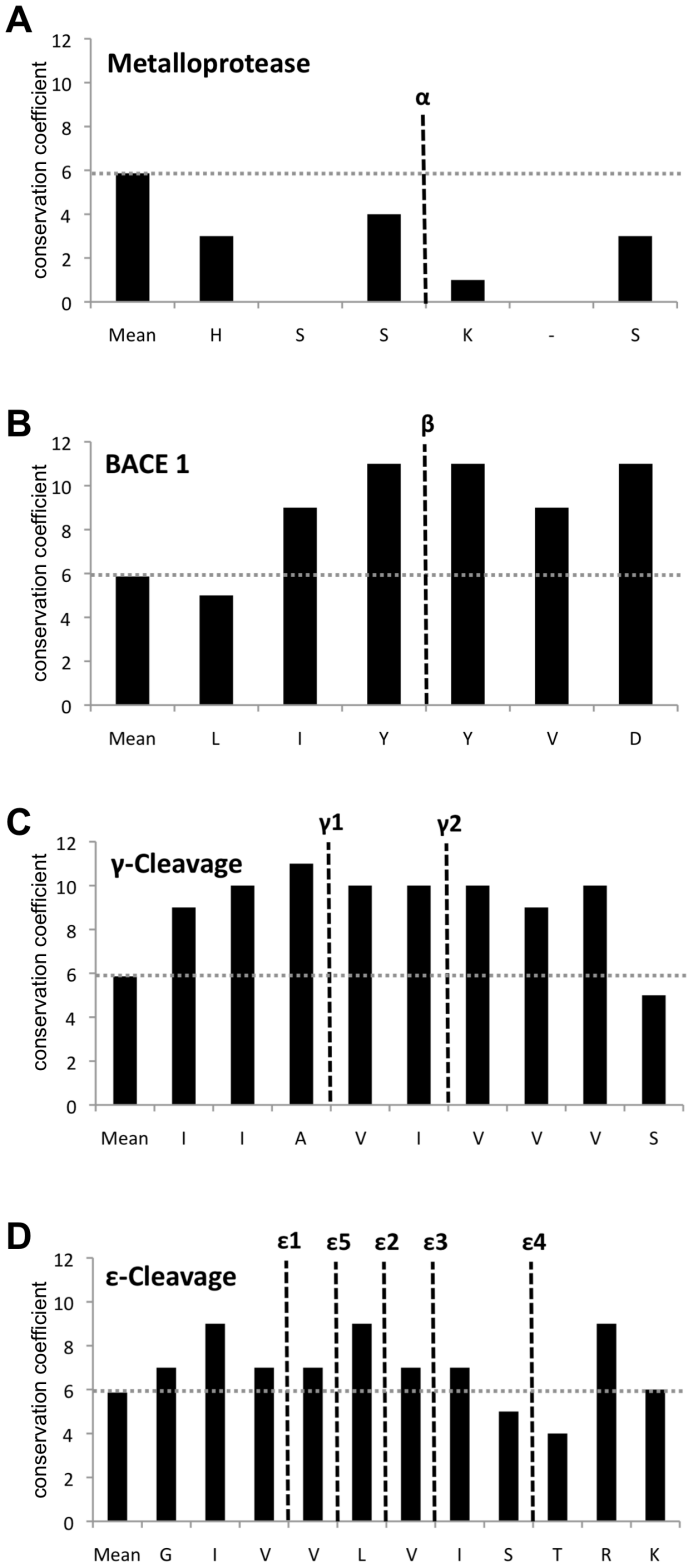
Sequence conservation of cleavage sites in orthologs of EpCAM found in fishes, amphibians, birds, to placental mammals. Amino acid sequences of 52 orthologs of human EpCAM were aligned using ClustallW and sequence conservation of each amino acid was calculated (maximum score 11. Shown are the mean conservation score throughout all orthologs (mean) and conservation scores of single amino acids ranging positions p^−3^ to p^+3^ around determined cleavage sites of metalloproteases (**A**), BACE1 (**B**), γ-cleavage of γ-secretase (**C**), and ε-cleavage of γ-secretase (**D**).

In 52 out of 61 sequences of potential orthologs, all exons encoding the mature EpCAM protein could be identified and were further considered to study the conservation of EpCAM cleavage sites (selected species are marked in bold letters in Table S2). It must be noted that exon 1, which encodes the signal peptide and part of the first glutamine within the mature protein, was identified in orthologs using SignalP 4.0 and disclosed from the following comparison (Figure S1). The conservation of cleavage sites within EpCAM across the 52 orthologs was assessed using the ClustalW algorithm with translated proteins sequences. Conservation coefficients were automatically calculated for each individual amino acid in the complete sequence and reached a value of 11 in case of a 100% identity throughout all species. The mean value of the conservation coefficient (CC) of the complete protein sequence of EpCAM across all 52 species was 5.86 and, thus, disclosed an intermediate 53% conservation of EpCAM in all orthologs (Figure 7). Cleavage sites were analysed in form of six amino acids from position P^−3^ to P^+3^ centred on the defined cleavage. The metalloproteinase cleavage sequence ^227^FHS*KKM^232^ in the extracellular domain of EpCAM (cleavage site *) displayed very low conservation below the protein average (CC 1–4) (Figure 7A), whereas the BACE1 cleavage site ^249^LIY*YVD^254^ was highly conserved (Figure 7B). Except for leucine in position P^−3^, all amino acids in the vicinity of the defined BACE1 cleavage site were characterised by higher-than-average conservation coefficient of 9 to 11 and both tyrosines, in between which cleavage occurs, have a conservation coefficient of 11, representing an almost perfect conservation throughout all species (Figure 7B). Cleavage sites of γ-secretase, which give rise to mEp-β fragments (γ-cleavage) also displayed very high sequence homology and a conservation coefficient of 9 to 11 (Figure 7C). In contrast, the conservation of cleavage sites of γ-secretase, which result in the generation of mEpICD fragments (e-cleavage), was more heterogeneous (CC 4–9). Cleavage ε2 was slightly more conserved in the leucine residue (CC 9) and cleavage ε4 was less conserved than the entire amino acid sequence of full-length EpCAM (CC 4–5) (Figure 7D).

Hence, BACE1 and g-cleavage sites within EpCAM displayed a great degree of conservation, whereas metalloproteinase and e-cleavage sites did not.

## Discussion

**Figure 8:**
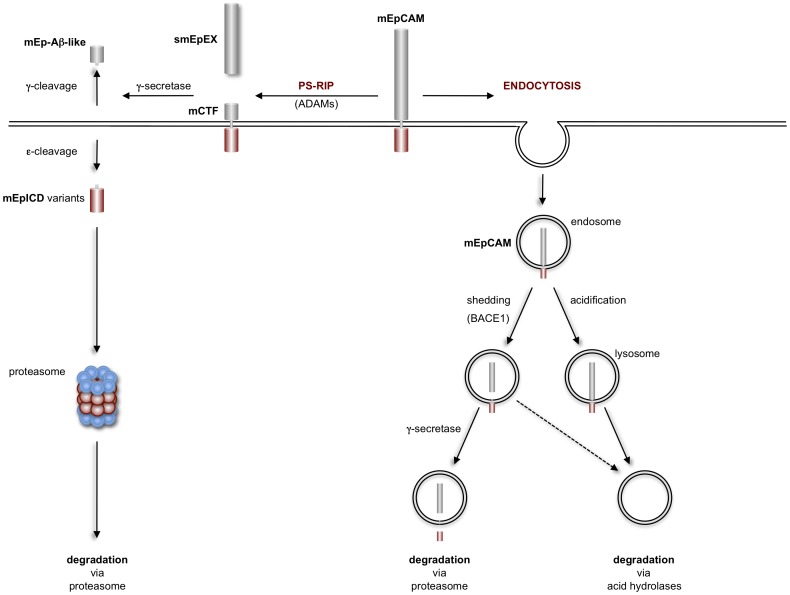
Schematic representation of EpCAM presenilin-dependent regulated intramembrane proteolysis (PS-RIP) and endocytosis. Murine EpCAM (mEpCAM) is cleaved at the plasma membrane to release soluble EpEX (smEpEX). The resulting C-terminal fragment (mCTF) is a substrate for γ-secretase, which cleaves mCTF to generate soluble, extracellular mEp-β fragments (γ-cleavage) and intracellular mEpICD fragments, which are prone to proteasomal degradation. Additionally, mEpICD can be endocytosed and processed either by BACE1 in acidic intracellular compartments (endosome) and/or by acidic hydrolases in lysosomes.

Regulated intramembrane proteolysis of EpCAM was first described in human carcinoma cells and after ectopic expression in HEK293 cells [10]. Further indications of a cleavage of human EpCAM were reported in human embryonic stem cells and in induced pluripotent stem cells [30], [31]. However, cleavage of orthologs of EpCAM has not been explored and the determination of precise cleavage sequences has been lacking entirely so far. Similarly to human EpCAM [10] and other substrates of regulated intramembrane proteolysis [1], murine EpCAM is subject to shedding through members of the metalloprotease family (see Figure 8 for a schematic representation). Additionally, mEpCAM and hEpCAM are newly identified substrates for the aspartyl protease BACE1, also termed β-secretase, which is central to the generation of the pathologic Aβ fragment of the amyloid precursor protein APP in Alzheimer’s disease [44], [45], [46], and might therefore also be involved in the production of mEp-β, itself a newly defined fragment of EpCAM (Figure 8). Despite a reported expression of BACE1 primarily in brain, hypothalamus, spinal cord, and pancreas [47], mRNA transcripts were detected in all cell lines used in the present study, with highest levels present in embryonic stem cells (data not shown). Fostered expression of BACE1 resulted in increased cleavage of both, human and murine EpCAM, and corroborated the results of treatments with small molecule inhibitors of BACE1. The intensity of cleavage of EpCAM through BACE1 was cell line-dependent and appeared most prominent in HEK293 cells, whereas it was minor in teratocarcinoma cells, where major cleavage of EpEX was essentially metalloprotease-dependent. In contrast, in fibroblasts an additional and yet to be characterised protease seems to have a major contribution to EpCAM shedding (see Figure 2B, NIH3T3 cells). Since BACE1 is active under acidic conditions and deploys its activity at a pH optimum of pH 4–4.5 [38], and primarily in endosomes and the trans-Golgi network [48], it is conceivable that differences in BACE1-dependent cleavage of EpCAM are related to differential targeting of EpCAM to endosomes.

Congenital tufting enteropathy (CTE) is a severe paediatric disease caused by a point mutation of EpCAM (Cys^66^Tyr). This mutant of EpCAM interferes with the proper localization into the cell membrane in several ways and eventually results in a lack of plasma membrane-associated EpCAM [49], [50]. However, the EpCAM (Cys^66^Tyr) mutant is still cleaved and is able to generate EpCAM-derived CTFs [49], [50]. Accordingly, it was speculated that EpCAM might be cleaved in the Golgi apparatus or in the ER lumen. Our data support the idea of a cleavage of EpCAM independently of its localisation at the plasma membrane and suggest cleavage to occur in intracellular compartments such as endosomes and lysosomes. It is tempting to speculate that BACE1 fulfils the cleavage of EpCAM (Cys^66^Tyr) mutant in CTE and might partially compensate for the missing EpICD generation at the cell membrane or prevent accumulation of the EpCAM (Cys^66^Tyr) mutant in endosomes. As deduced from cleavage assays performed at different pH values, cleavage of mEpCAM at the plasma membrane (pH 7) appears far less efficient than in acidified intracellular organelles (pH4). From this, we suggest that only a proportion of EpCAM molecules are cleaved at the plasma membrane, whereas EpCAM is cleaved to nearly 100% once internalised in endosomes. Quantitative cleavage of mEpCAM in the trans-Golgi network on its way to the plasma membrane appears implausible since mEpCAM can be detected at the plasma membrane to high levels with antibodies binding within the ectodomain. Anyhow, it appears that metalloproteases and BACE1 are the major sheddases involved in regulated intramembrane proteolysis of EpCAM, although we cannot rule out that other proteases might play important roles in the shedding of murine EpCAM.

We determined several cleavage sites within the intramembrane domain of mEpCAM, which gave rise to newly identified Ab-like fragments now termed mEp-β and mEpICD variants (Figure 8). The existence of Aβ-like fragments of mEpCAM is described for the first time in the present study and might add up to a potential role for this part of the protein, possibly in conjunction with the cleavage through BACE1, as was extensively described for APP in Alzheimer’s disease [46]. Gelatinous drop-like corneal dystrophy (GDLD) is an autosomal recessive disorder characterized by corneal amyloidosis leading to blindness. GDLD is associated with mutations in the EpCAM paralog Trop2, also known as TACSTD2 [51], which was most probably generated through retroposition of the *EpCAM* transcript [52]. Although speculative in nature, it is conceivable that regulated intramembrane proteolysis of Trop2, which was described recently in detail [37], results in the generation of Aβ-like fragments similarly to EpCAM and contributes to corneal amyloidosis. Described mutations in GDLD are located away from potential cleavage sites within Trop2, however, these changes in the amino acid composition might impact on the overall structure and, thus, indirectly on processing of the protein. Owing to two different γ-cleavage sites and to leastwise one α- and one β-cleavage site, a combinatorial of four different Aβ-like fragments of EpCAM can be envisaged. A potential role for mEp-β fragments from EpCAM is so far unclear and subject to further studies.

Out of the five different mEpICDs variants determined in the present study, only one fragment was stable enough to be detected at decent levels in mass spectrometry of untreated cells and might represent the mEpICD protein detected to low amounts in immunoblot experiments. This mEpICD molecule displayed an N-terminal region composed of valine and isoleucine, while an mEpICD fragment with the amino acids valine and leucine added to its N-terminus displayed greatly diminished stability. Since the first amino acid at the N-termini of both EpICD variants were identical (valine), differences in stability can hardly be explained according to the N-end rule. Furthermore, mutation of either the leucine or the isoleucine in second position of the mEpICD variants did not significantly alter stability (*data not shown*). Interestingly, both mEpICD fragments were generated in similar amounts at the plasma membrane as demonstrated in membrane-based assays. The observed decreased expression of the longer mEpICD species in lysates was was dependent upon the proteasome. Furthermore, inhibition of γ-secretase induced an accumulation of the murine and human CTF fragments of EpCAM, which were only marginally increased upon further treatment with proteasome inhibitors and which might be the result of endoplasmatic-reticulum-associated protein degradation (ERAD) [53]. Based on these findings and on the fact that treatments with γ-secretase and proteasome inhibitors were not additive, we conclude that EpICDs but not CTFs are subject to differential proteasomal degradation. Ectopically expressed variants of mEpICD, which mimic endogeneous EpICDs, confirmed these assumptions. Differential stability of intracellular domains was similarly demonstrated for the case of Notch [54]. Here, stability of Notch-ICD impacted on the signalling capacity of the Notch receptor with a substantial silencing through degradation. Furthermore, endocytosis was implicated in the regulation of the generation of differing Notch-ICD variants. Together with the fact that BACE1 is active in the acidic milieu of endosomes [38], it is tempting to speculate that BACE1-mediated cleavage of EpCAM in endosomes results in the formation of mEpCID molecules with differing stability and contributes to the regulation of the overall amount of EpCAM in cells in a very efficient manner. It must however be noted that the overall stability of murine EpICD variants was anyhow very low and proteasomal degradation of mEpICD very potent. Human EpCAM is a rather stable protein with a half-life of 21 hours at the plasma membrane [55]. However, in certain conditions rapid down-regulation of EpCAM might appear necessary, for example in cells undergoing differentiation such as tissue progenitors and embryonic stem cells, in which rapid and efficient fine-tuning of EpCAM expression might be of paramount importance [30], [32], [56]. In such situations, regulated intramembrane proteolysis, which induces a quantitative degradation of EpCAM (via EpICD degradation), might support or even precede effects at the transcriptional levels.

In homology to murine EpCAM, we have confirmed the existence of C-terminal fragments of human EpCAM and demonstrated a comparable sensitivity of hEpICD towards proteasomal degradation, which corroborated the effective degradation of hEpICD first mentioned in 2009 [10]. The existence of human CTF, whose generation precedes that of hEpICD, is in accordance with the findings by Schnell *et al.*, who reported on the existence of hEpCAM CTFs of 20 kD and ranging from 12 to 15 kD [49]. Although we could not confirm the presence of a 20 kD CTF at all time points of cleavage, human and murine CTFs ranging from 12 to 15 kD were detected in HEK293 cells, and murine CTFs were described at the single amino acid levels using mass spectrometry approaches. A mimic of hCTF, which lacked biggest parts of the extracellular domain of wildtype hEpCAM, was quantitatively processed to hEpICD and thus far more efficiently cleaved by the γ-secretase complex than wild-type hEpCAM. Hence, the initial shedding of human and murine EpCAM represents the rate-limiting step in the cleavage of the protein and most probably dictates cleavage levels as seen at the plasma membrane.

Lastly, the conservation of cleavage sites, and thus the potential importance of EpCAM cleavage, was addressed in newly identified orthologs of the protein. Sequences corresponding to the full-length protein or parts of EpCAM were identified in a total of 61 different species ranging from fishes, amphibians, birds, to placental mammals. Fifty-two orthologs could be assigned unequivocally and were considered in the comparative study. Eventually, BACE1 and the γ-cleavage site displayed a very high degree of conservation throughout all orthologs, whereas metalloprotease and ε-cleavage sites displayed very low and an intermediate conservation, respectively. This very high degree of conservation in BACE1 and g-cleavage sites were also seen in incomplete sequences of the most distant ortholog in the lamprey, a jawless fish of a very ancient lineage of vertebrates. Since BACE1 and the γ-cleavage site have been conserved to such a high degree, it is imaginable that the generation of mEp-β fragments is of major importance for the function of EpCAM throughout evolution.

In summary, we present a comprehensive overview of cleavage processes associated with the tumour and stem cell associated receptor EpCAM at the single amino acid level. Novel aspects relate to the shedding of EpCAM through BACE1 most probably after endocytosis of the molecule, the generation of Ab-like fragments, and the existence of various mEpCID molecules that are efficiently but differentially degraded via the proteasome (Figure 8). According to similarities at the amino acid level, these features might be shared by orthologs of EpCAM and Trop2 proteins in numerous vertebrates and therefore might represent a general theme of regulation of EpCAM.

## Materials and Methods

### Cell Lines

E14TG2a embryonic stem cells [57] were a kind gift from Dr. Marcus Conrad (Munich) cultured in Stempan Gmem medium (PAN-Biotech, Aidenbach, Germany) and leukemia inhibitory factor (LIF, 1,000 U/ml; Merck KGaA, Darmstadt, Germany) on 0.1% gelatin-coated 6-well plates. Murine F9 teratoma (kind gift from Dr. Marcus Conrad, Munich) cells were cultured in Dulbecco’s modified Eagle’s medium (DMEM, high glucose) supplemented with 20% FCS (Biochrom AG, Heidelberg, Germany) and 1% penicillin/streptomycin. Human embryonic kidney 293 cells (HEK293) and NIH3T3 fibroblasts were cultured in DMEM supplemented with 10% FCS and 1% penicillin/streptomycin. All cell lines were grown in a 5% CO_2_ atmosphere at 37°C.

### Transfections and Expression Vectors

Transfections were performed with the MATra reagent (Iba, Goettingen, Germany) following the manufacturer’s recommendations. Alternatively, embryonic stem cells were transfected with the Amaxa Nucleofector system and the Mouse ES Cell Nucleofector Kit (Lonza, Ratingen, Germany). EpCAM full-length (314 aa) was cloned in fusion with a YFP-Tag to generate EpCAM-YFP. The EpCAM-TF construct was cloned by the introduction of a TEV-cleavage site (ENLYFQG) followed by a FLAG-tag (DYKDDDDK) between amino acid 223 and 224 of EpCAM. Myc-CTF-FT-YFP consists of the signal peptide of murine EpCAM (1–23), a short linker peptide consisting of two amino acids (KL), a Myc-Tag (EQKLISEEDLYVDEKAPEFSMQGLT), the CTF sequence of murine EpCAM (251–315), a Flag-Tag (DYKDDDDK), and the TEV recognition site (ENLYFQG) followed by a YFP-Tag. Expression vectors for the EpICD mutants MVLVI-EpICD-YFP (251 to 315 aa) and MVI-EpICD-YFP (253 to 315 aa) are N-terminal truncated versions of the cMyc-CTF-FT-YFP construct. All constructs mentioned above were cloned into the 141 pCAG-3SIP expression vector by using EcoRI and NheI restriction enzyme sites. Stable selection of transfectants was performed with puromycin (4 ng/ml) in the according culture medium starting at one day after transfection. The pcDNA3.1 plasmid was used for BACE1 over-expression. For determination of the α- and β-cleavage sites transient BACE1 over-expression was performed 24 h before the medium was changed.

### Membrane-based EpCAM Cleavage Assay

These assays were performed as described earlier [34].

### Protein Analysis

For immunobloting, cells were lysed in PBS containing 1% n-dodecyl β-D-maltoside (Sigma, Munich, Germany) and protease inhibitors (Roche complete, Roche, Grenzach, Germany). Protein concentrations were determined using the BCA-assay (Thermo Scientific, Erlangen, Germany). Equal amounts of proteins were separated by SDS-PAGE (10–15%) and visualized using antibodies against mEpICD (immunising peptide:TRKKSAKYEKAEIKEMGEIHRELNA,Charles River, Cologne, Germany), hEpICD and YFP (anti-GFP antibodies bind specifically to YFP; Santa Cruz, Heidelberg, Germany) in combination with horseradish peroxidase (HRP)-conjugated secondary antibodies, and the ECL reagent (Millipore, Darmstadt, Germany). Bioluminescence was assessed in a Chemidoc XRS+ imaging system (Bio-Rad, Munich, Germany).

### Proteomics and Mass Spectrometry

#### α- and β-cleavage sites

After cells were grown to confluency, medium was changed and collected after 24 h for Flag IPs. Supernatants from NIH3T3 and HEK293 cell lines (15 ml) and mF9 cell line (50 ml) were used for the Flag immunoprecipitation with 30 µl of Flag beads (M2 Sigma, Munich, Germany) over night at 4°C on a rotating device. Beads were washed 3 times with PBS and twice with water. Proteins were eluted with 40 µl glycine (100 mM; pH 2,5) on ice for 15 min. Eluted peptides were transferred to a new vial and neutralized by addition of 200 µl Tris (100 mM; pH 8). Overnight digestion with TEV protease (Invitrogen, Cologne, Germany) was performed following the manufacturer’s recommendations. Flag-Tag containing peptides were immunoprecipitated with 10 µl Flag-beads at 4°C for 4 hours in a rotating device. After washing of beads 3 times with PBS and twice with water, peptides were eluted in 10 µl acetonitrile (Sigma, Munich, Germany) and water (1∶1) saturated with α-cyano-4-hydroxyl-cinnamic acid (Sigma, Munich, Germany), and subsequently analysed in a Voyager-DE STR mass spectrometer (Applied Biosystems, Cologne, Germany).

#### γ-cleavage sites

After cells were grown to confluency, the medium was changed and collected after 24 h for Myc-immunoprecipitation. For this immunoprecipitation, 50 ml of cell culture supernatant and 15 µl of Myc-beads (Sigma, Munich, Germany) were mixed overnight in a rotator device at 4°C. The washing steps and peptide elution were performed as described above.

#### ε-cleavage sites

Immunoprecipitation was performed with the YFP-Trap system (Chromotek, Munich, Germany) using 4000 µg protein lysate in combination with 30 µl YFP-Trap at 4°C for 4 h in a rotating device. All following steps were performed as described above.

Cells stably expressing cMyc-CTF-FT-YFP used for membrane isolation were treated for 24 h with DAPT with the concentration of 1 µM (Sigma, Munich, Germany).

### Inhibitors

Inhibition of α-, β- and γ-secretase was performed using TAPI-1 (50 µM, Merck-Millipore, Darmstadt, Germany), C3 (1 µM, Merck-Millipore, Darmstadt, Germany), and DAPT (1 µM, Sigma, Munich, Germany) respectively. Inhibitors were supplemented when medium was changed. Phorbol ester phorbol-12-myristate-13-acetate (Sigma, Munich, Germany) was substituted in a concentration of 1 µM, 2 h before the cell culture supernatant was collected. Treatment with lactacystin β-lactone (Santa Cruz, Heidelberg, Germany) with a concentration of 50 µM was performed for 12 h. MG132 (Merck-Millipore, Darmstadt, Germany) was supplemented 4 h before cells were harvested into the medium with a final concentration of 10 µM.

### Flow Cytometry

YFP expression of cells was analysed in a FACScalibur cytometer (Becton Dickinson). FACS buffer contained 3% of FCS in PBS. Living cells were gated according the forward (FSC) and side scatter (SSC).

### RNA Isolation, cDNA Synthesis and Quantitative Real-time Polymerase Chain Reaction

RNeasy Plus Universal Kit (Qiagen, Hilden, Germany) was used for RNA isolation. For cDNA synthesis, QuantiTect Reverse Transcription Kit (Qiagen, Hilden, Germany) was used. The QuantiTect SYBR Green PCR Kit (Qiagen, Hilden, Germany) was used for the measurement in a light cycler 480 (Roche, Mannheim, Germany).

### Immunoprecipitation of hEpEX

Immunoprecipitation of hEpEX was performed as described earlier [10].

### Identifications of EpCAM Orthologs in different Species and Alignment of Corresponding Sequences

Various amino acid sequences from orthologs of EpCAM were retrieved from available databases in NCBI blastp and UniProt with the amino acid sequence of human EpCAM (NP_002345.2) as bait. Additional EpCAM sequences could be identified by BLASTN using all exon sequences of human EpCAM for the search in the Ensembl database. The software Jalview and the ClustalW Multiple Sequence Alignment algorithm were used for alignments and calculation of conservation values.

## Supporting Information

Figure S1 **Sequence alignment of EpCAM amino acids including localisation of the predicted transmembrane domain (TMD), α, β, γ, and ε cleavage sites.**
(DOCX)

Table S1 **Comparison of ADAM and BACE1 cleavage sites in described substrates. m: murine; h: human, aa: amino acids.**
(DOCX)

Table S2 **Vertebrate EpCAM amino acid sequence comparisons with the Homo sapiens EpCAM sequence.** Latin species names (bold, full length sequences of mature EpCAM proteins available), abbreviations used, common names, accession numbers with hyperlink, amino acid identities, similar amino acid position in % are listed. (n.a. not applicable because sequences are incomplete).
(DOCX)
