# Supplementary material for: Regulated Intramembrane Proteolysis and Degradation of Murine Epithelial Cell Adhesion Molecule mEpCAM
Source: PLoS One. 2013 Aug 29;8(8):e71836. doi: 10.1371/journal.pone.0071836 (PMC3756971; doi:10.1371/journal.pone.0071836)
Supplement: Figure S1 — Sequence alignment of EpCAM amino acids including localisation of the predicted transmembrane domain (TMD), α, β, γ, and ε cleavage sites. (DOCX) [file pone.0071836.s001.docx]

10 20 30 40 50 60 70 80 90 100 110 120 130 140 150 160 170

| | | | | | | | | | | | | | | | |

Mpu_EpCAM -----XXCVCENYKLTTNCSLNAHGQCECTSLGA---QNSVICSK---LATKCLVMKAEMTSS-KSGRRVRP--------EGAFQNNDGLYDPDCDEKGLFKAKQCNGTTTCWCVNTAGVRRTDKDNEEEISCSERVRTYWIIIELKHKTRET--PYDIESLRTALKEII

Oro_EpCAM ----LTPCVCDKYKLTTNCSLNANGLCECTSLGA---QNSVICSK---LATKCLVMKAEMTRS-KSGRRGRP--------EGAFQNNDGLYDPDCDEKGLFKAKQCNGTTTCWCVNTAGVRRTDKDNDDEISCSERVRTYWIIIELKHKTRET--PYDTESLRTALLKTI

Ame_EpCAM -----XXCVCENYKLTTNCSLNTYGQCECTSLGT---QNSVICSK---LATKCLVMKAEMTSS-KSGRRVKP--------EGAFQNNDGLYDPDCDEKGLFKAKQCNGTATCWCVNTAGVRRTDKD--AEITCTERVRTYWIIIELKHKTRET--PYDMESLKTALKDAI

Cfa_EpCAM ----XXACICENYKLTTNCSLNINNQCECTSIGA---QNSVICSK---LATKCLVMKAEMTGT-KSGRRARP--------EGAFQNNDGLYDPDCDEKGLFKAKQCNGTTTCWCVNTAGVRRTDKD--TEISCTERVRTYWIIIELKHKTRET--PYDTQSLQNALKETL

Fca_EpCAM ----QKGCVCENYKLTTNCSLNTHGQCECTSIGA---QNSVICSK---LATKCLVMKAEMTGT-KSGRRARP--------EGAFQNNDGLYDPDCDEKGLFKAKQCNGTTTCWCVNTAGVRRTDKD--TEISCSERVRTYWIIIELKHKTRET--PYDIKSLQTALKEVI

Bta_EpCAM ----QEGCVCENYKLTTNCSVNALGQCQCTSVGT---QHSVICTK---LATKCLVMKAEMNHS-KSGRRGKP--------EGAIQNNDGLYDPECDDKGLFKAKQCNGTSTCWCVNTAGVRRTDKD--SEISCSEPVRTYWIIIELKHKTREK--PYDLQSLQSALKDVI

Oar_EpCAM ----QEGCVCENYKLTTNCSVNALGQCQCTSVGT---QHSVICTK---LATKCLVMKAEMSHS-KSGRRRKP--------EGAIQNNDGLYDPECDDKGLFKAKQCNGTSTCWCVNTAGVRRTDKD--SEISCSEPVRTYWIIIELKHKTREK--PYDLQSLQSALKEVI

Oor_EpCAM ----QEGCVCENYKLTTNCSVNAHGQCQCTSIGT---QHSVICTK---LASKCLVMKAEMSGS-KAGRRVKP--------EGAIQNNDGLYDPECDDKGLFKAKQCNGTSTCWCVNTAGVRRTDKD--SEISCSEPVRTFWIIIELKHKTREK--PYDVQSLQAALKEVI

Ttr_EpCAM ----QEGCVCENYKLTTNCSVNAHGQCQCTSIGT---QHSVICTK---LASKCLVMKAEMSGS-KAGRRVKP--------EGAIQNNDGLYDPECDDKGLFKAKQCNGTSTCWCVNTAGVRRTDKD--SEISCSEPVRTFWIIIELKHKTREK--PYDVQSLQAALKEVI

Ssc_Epcam ----QQGCVCENYKLTTNCSLNALGQCQCTSIGA---QNSVICSK---LASKCLVMKAEMTGS-KAGRRLKP--------ENAIQNNDGLYDPDCDENGLFKAKQCNGTSMCWCVNTAGVRRTDKD--SEISCLERVRTYWIIIELKHKTREK--PYDVTSLQNALKEVI

Vpa_EpCAM -----KDCVCENYKLTTNCSLNAYGQCQCTSLGT---QNSVICSK---LATKCLVMKAEMTVS-KDGRKKKP--------EGAIQNNDGLYNPDCDEKGLFKAKQCNGTSMCWCVNTAGVRRTDKD--SEISCSEQVRTYWIIIELKHKAREK--PYDVQSLQTALKEVI

Eca_EpCAM ----QKDCICENYKLTTNCSVNMYGQCQCTSLGT---QNSVICSK---LASKCLVMKAEMTHS-KSGRRVKP--------EGAIQNNDGLYDPDCDEKGLFKAKQCNGTAMCWCVNTAGVRRTDKD--SEISCLERVRTYWIIIELKHKTREK--PYDVQSLQNALKETI

Mlu_EpCAM ---QEGECICENYKLTTNCSTDTYGQCRCTSIGT---QHTVICSK---LATKCLVMKAEMSHS-KSGRRPKP--------EDAIQNNDGLYDPDCDENGHFKAKQCNGTTTCWCVNTAGVRRTDKD--TEKTCSERVRTYWIIIELRHKTREK--PYNSQSLQTALKEII

Laf_EpCAM ---QKVDCICEKYKLTTNCSTNWNGQCQCTSIGT---QNTVICSK---LATKCLVMKAEMTTS-KAGRRVKP--------EGAIQNNDGLYDPDCDEKGLFKAKQCNGTATCWCVNTAGVRRTDKD--TEIMCTERVRTYWIIIELKHKTRDK--PYDLQSLRTALEKAI

Ppa_EpCAM ----QEECVCENYKLAVNCFVNNNHQCQCTSIGA---QNTVICSK---LAAKCLVMKAEMNGS-KLGRRAKP--------EGALQNNDGLYDPDCDESGLFKAKQCNGTSTCWCVNTAGVRRTDKD--TEITCSERVRTYWIIIELKHKAREK--PYDGKSLRTALQKEI

Ptr_EpCAM -----XXCVCENYKLAVNCFVNNNHQCQCTSIGA---QNTVICSK---LAAKCLVMKAEMNGS-KLGRRAKP--------EGALQNNDGLYDPDCDESGLFKAKQCNGTSTCWCVNTAGVRRTDKD--TEITCSERVRTYWIIIELKHKAREK--PYDGKSLRTALQKEI

Ggo_EpCAM ----QEECVCENYKLAVNCFVNNNRQCQCTSIGA---QNTVICSK---LAAKCLVMKAEMNGS-KLGRRAKP--------EGALQNNDGLYDPDCDESGLFKAKQCNGTSTCWCVNTAGVRRTDKD--TEITCSERVRTYWIIIELKHKAREK--PYDGKSLRTALQKEI

Hsa_EpCam ----QEECVCENYKLAVNCFVNNNRQCQCTSVGA---QNTVICSK---LAAKCLVMKAEMNGS-KLGRRAKP--------EGALQNNDGLYDPDCDESGLFKAKQCNGTSMCWCVNTAGVRRTDKD--TEITCSERVRTYWIIIELKHKAREK--PYDSKSLRTALQKEI

Nle_EpCAM ----QEECVCENYKLAVNCFVNNNGQCQCTSIGA---QNTVICSK---LAAKCLVMKAEMNGS-KLGRRAKP--------EGALQNNDGLYDPDCDESGLFKAKQCNGTSTCWCVNTAGVRRTDKD--TEITCSERVRTYWIIIELKHKAREK--PYDGKSLRTALQKEI

Mml_EpCAM ----QKECVCENYKLAVNCFLNDNGQCQCTSIGA---QNTVLCSK---LAAKCLVMKAEMNGS-KLGRRAKP--------EGALQNNDGLYDPDCDESGLFKAKQCNGTSTCWCVNTAGVRRTDKD--TEITCSERVRTYWIIIELKHKAREK--PYDVQSLRTALEEAI

Pan_EpCAM ----QKECVCENYKLAVNCFLNDNGQCQCTSIGA---QNTVLCSK---LAAKCLVMKAEMNGS-KLGRRAKP--------EGALQNNDGLYDPDCDESGLFKAKQCNGTSTCWCVNTAGVRRTDKD--TEITCSERVRTYWIIIELKHKAREK--PYDVQSLRTALEEAI

Cja_EpCAM ----XXECHCENYKLAINCVMNSNGQCQCTSVGS---QNTVICSK---LASKCLVMKAEITNS-KLGRRAKP--------EGALQNNDGLYDPDCDESGLFKAKQCNGTSTCWCVNTAGVRRTDKD--TEITCSERVRTFWIIIELKHKAREK--PYDVQSLRDALQEAI

Ocu_EpCAM ----QENCICKNYKLTTNCVEK-NGDCQCTSVGT---QNTVLCSK---LASKCLVMKAEMTSS-KSGRRVKP--------EGAIQNNDGLYDPECDEQGLFKAKQCNGTATCWCVNTAGVRRTDKD--TEIMCSERVRTYWIIIELKHKSREK--PYDTQSLQTALQELI

Mmu_EpCAM ----QRDCVCDNYKLATSCSLNEYGECQCTSYGT---QNTVICSK---LASKCLAMKAEMTHS-KSGRRIKP--------EGAIQNNDGLYDPDCDEQGLFKAKQCNGTATCWCVNTAGVRRTDKD--TEITCSERVRTYWIIIELKHKERES--PYDHQSLQTALQEAF

Rno_EpCAM ----QKDCVCNNYKLTSRCYENENGECQCTSYGT---QNTVICSK---LASKCLVMKAEMTHS-KSGRRMKP--------EGAIQNNDGLYDPECDEQGLFKAKQCNGTATCWCVNTAGVRRTDKD--TEITCSERVRTYWIIIELKHKERAQ--PYNFESLHTALQDTF

Cgr_EpCAM ----XXXCVCENYKLGTSCSLNDRGECQCTSLGT---SNTVVCSK---LASKCLVMKAELTYSNKSGRRLKP--------EGAIQNNDGMYNPECDELGLFKAKQCNGTATCWCVNTAGVRRTDKD--TEITCSERVRTYWIIIEIKHKERES--PYNLQNLKTALEEVF

Cpo_EpCAM ----QPTCICENYRMTTNCFLD-NGQCKCTAYGT---QNSVLCST---LASKCLVMKAEMTQS-KSGRRAKP--------EGAIQNNDGLYDPECDEHGRFKAKQCNGTSTCWCVNSAGVRRTNKD--SEIQCSETVQTYWIIIELKHKERKQ--PFDLQSLQAAIQEVL

Mdo_EpCAM ----QEACYCETNKLTNCTNIP-FEGCQCHAIGS---SHLITCSK---IASKCLIMKAEMLNS-KMGRRKKP--------EGAIQDNDGIYDPDCDIHGHFKPRQCNGTALCWCVNTAGVRRTDKE--TEIACDELVRTYWIIIELKHKTREK--PFNTTILENALKEEF

Sha_EpCAM ----GKACICETNKLTNCTEIS-PDECQCQAIGS---PNIVTCST---LATKCLLMKAEMTNS-KSGRRKRP--------EGSFKDNDGLYDPDCDTHGHFKPRQCNGTALCWCVNTAGVRRTDKD--TEISCSELVRTYWIGIELKHKTREK--PFDSTILKNALIKEF

Cad_EpCAM ----QEECVCEKNKRTTDCSVFND-VCQCKSIGF---NITVNCQT---LTSKCLLMKAEVAGLK-SGRRKRP--------EHAYVDNDGIYDPECDAKGNFKPKQCNGTKTCWCVNSAGVRRTEKS-DQNITCQEVVRTSWIIIETKCRDGNT--HPDKASLERTIEKLF

Psi_EpCAM ----KIACSCATNKRTTNCSMSSG-TCQCTSFGS---QVLVNCQT---LTSKCLLMKAEMAALKKSGRRERP--------KHAYVDNDGIYDPDCENSGKFKARQCNGT-SCWCVNTAGVRRTEKS-DADLQCSELVRTSWILIETQHGERSK--DVDVSQLKETIKNEF

Gga_EpCAM ----QDSCTCTKNKRVTNCKLIDN-VCHCNSIGS---SVSVNCEI---LTSKCLLMKAEMANTK-SGRREKP--------KDALQDTDGLYDPECENNGLFKAKQCNGT-TCWCVNTAGVRRTDKH-DTDLKCNQLVRTTWIIIEMRHAERKT--PLNAESLTRYLKDTI

Mga_EpCAM ----XXPCTCTKNKRVTNCRMIDG-VCNCNSIGS---SVSVNCET---LTSKCLLMKAEMSNTK-SGRREKP--------KDAFQDTDGLYDPECENTGVFKAKQCNGT-TCWCVNTAGVRRTDKH-DTDLKCNQLVRTMWIIIEMKHTERKT--PLKAESLTKLLKDTI

Tgu_EpCAM ----XXACICEKNKRVSNCRMDSSGQCRCEAIGS---GVTVDCST---LTSKCLLMKAEVMGSK-SGRREKP--------KDAFEDTDGLYDPECENNGTFKAKQCNGS-TCWCVNTAGVRRTDKH-DTDLKCNHLVRTMWIIIEMKHAERNA--PLNAESLKKFFTDTI

Aca_EpCAM ----QDGCICPMN-IRTTCEME-SGSCICRVLGS---NQRVNCST---LTSKCLLMKAEMYRT--PRRFPKP--------EHAFLDNDGLYDPDCDASGIFKARQCNKTDTCWCVNSAGVRRTDKG-DDNMRCSELVRTNWILIELKQKERVD--PFLEAEVENSLRQFI

Xla_EpCAMa ---QSQGCKCRTHYMGKCDNSGASSDCQCTLTIGPD-SQPVNCSK---LIPKCWLMKRESLGT-KAGRRVKP--------AQALIDNDGLYNPECDTNGVFKARQCNNTDTCWCVNTAGVRRTDKG-DKNWKCPELVRTNWVYVEMKRNNTDS--VND-DDLKKALKTTI

Xla_EpCAMb ---QNPGCKCKTHYLGKCDNSGASSDCQCALSIGPA-SQAVDCTK---LIPKCWLMKRESLGT-KAGRRVKP--------VQALIDNDGLYDPECETNGVFKARQCNNTDTCWCVNTAGVRRTDKG-DKNWKCPELVRTNWVIVEMKRNNSDS--VND-DVLIQALKTTI

Xtr_EpCAM ---QSPGCTCSTLYMGKCDNSGAG-GCQCTLAIGTA-TQSINCSA---LIPKCWLMKRESLGT-KAGRRVKP--------VQALVDNDGLYDPECDVNGVFKARQCNNTDTCWCVNSAGVRRTDKG-DKNWKCPELVKTNWVIVEMKRNGTES--VSD-ADLIQALKTTI

Aam_EpCAM ---QNCPNSCETMKWATCDGPP----CSCYLLVGDNQKQPLNCAA---LIPKCFLMKAEMYRA-KKGLDTR--TVGGKPVETAFVDNDGIYDPECEADGKFKAKQCNNTEECWCVNSAGVRRSDKG-DKNIKCEKLVETYWVRLQLTHKDGAPK-SLSESKLKDAIADAL

Asa_EpCAM ---QNCPNSCETMKWATCDGTP----CSCYLLVGDNQKQPLNCAA---LIPKCFLMKAEMYRA-KKGLDTR--TVGGKPVETAFVDNDGIYDPECEADGKFKAKQCNNTEECWCVNSAGVRRSDKG-DKNIKCEKLVETYWVRLQLTHKDGAPK-SLSESKLKDAIADAL

Oni_EpCAMa ---QICQSTCETMKWATCDGPP----CSCYLLVDNGVKQPINCTT---LVPKCFLMKAEMYRA-RKGMNTR--SIAGKPDETAIVDNDGIYDPECESDGKFKAKQCNGTDVCWCVNSAGVRRTDKG-DQTLKCEKLVETHWIRLQLTHKQGPA--TVDKDKLKTAISTEL

Oni_EpCAMb ---QSCPSTCETMKWGTCDGPP----CSCYLLVDNGVKQPIDCNA---LVPKCFLMKAEMYRA-RKGMSTR--SIAGKPDETAIVDNDGIYDPECESDGKFKAKQCNGTDVCWCVNSAGVRRTYKG-DQTLKCEKLVETYWIRLQLTHKQGP---TVTKDKLKTAIVAEL

Tru_EpCAM ---QSCS--CETMKWATCDGTP----CSCFVLVDGGVKQTLECDK---LIPKCFLMKAEMYRA-RKNLDTR--TIGGKPVETAFVDNDGIYDPDCENDGKFRAKQCNNTDECWCVNSAGVRRTDKG-DKDLKCEKLVETHFVRLQLTHKPTPN--PVKAADLKTAIAEAI

Tni_EpCAM ---QACS--CETMKWATCDGNP----CGCYLLVNNGEQQKVDCTKSTPVIPKCYLMKAEMYRA-RKNLDTRS-TLGGKPVETAFVDNDGIYDPECENDGKFRAKQCNNTEKCWCVNSAGVRRTDKG-DKDLKCEKLVETHFVRLQLTHKETPQ--PVDATGLKTAIADAI

Gac_EpCAM ---QSCS--CETMKWATCDDAP----CACNILTGDNNRQPLNCTS---LIPKCWLMKAEMYRA-KKGLDTR---TGGKPVESAFVDNDGIYDPECQSDGKFKARQCNNTEECWCVNSAGVRRTDKG-DKTLVCEKLVETHWLRLQLTHKALTT--PLKAAELKDAIADAI

Omo_EpCAM ----QCPQSCESLKWATCDGPP----CQCTIIFGDDVKQPLTCDK---LIPKCFLMKAEMYRS-RKGLSTRT-GIGGKPVETAFVDNDGIYDPECENDGKFKAKQCNSTEECWCVNSAGVRRSDKG-DKDIKCEKLVETYWIRLQLKHKEVST--SVDKDQLKSAIGTAI

Cid_EpCAM ----QCI--CNSMKWAKCDGSP----CQCTLQFTDEIQQPLDCTK---LVPMCYLMKAEMYRA-KKNLSTRSI--GGKPVETAFVDNDGIYDPECENDGKFKAVQCNNTDVCWCVNSAGVRRSDKG-DKSIKCEP-VETYWVRLELKHKATDV--PLDADKLKTGIQNAL

Dre_EpCAM ----QCA--CKTMKWANCDDS-----CSCSLRLTESSTQTLNCSK---LVPKCFLMQAEMYRA-RNHQDTRS---GGKPVETAFVDNDGIYDPVCESDGKFKAVQCNNTEVCWCVNSAGVRRSDKK-DKNIKCEP-AETYWVRVEMKHKSVDV--PIDATKLRTGIENVL

Ola_EpCAM ---QECQ--CDTVKWASCTGPP----CQCTIPLTKDINQPLNCNA---LAPKCFLMKTEMLRR-SKGQDTR---TVGKP-EEGFVDNDGIYNPECENDGKFKAKQCNNTEECWCVNSAGVRRTEKE-GPNINCDKLVETFWIRLILEHAPLQG--AVDKNLLKSALGDAI

Xma_EpCAM ----ACS-TCRTMKWVNCEGAAPP--CTCQITLDDSNRPAIDCEK---LVSKCFLMKAEMYRR-KMGQDVRT-NIGGKPHEDAIMDNDGIYNPDCENDGKFKAKQCNNTEECWCVNSAGVRRTDKG-DKNMNCSKLVETYMIRLELTHKELESNNKVNVQALENSVKNLL

Cmi_EpCAM QTDEACAP-CTTNRYARCSGTP----CQCTLKISKSEAIPVNCSI---MTRKCWMMKAEMYRI-VNGVGTR-----RKPSEDALLDTDGIYDPECNDVGDFHAKQCNGTDVCWCVNSAGVRRTDKG-DTDKKCDELVKTFWFRIELRHKPVDS---IDENKLIEKIT-KV

* * * : * : * *: * : . :.**:*:* *: * *:. *** : *****:*****: * * ..* . : : . .

TMD

180 190 200 210 220 230 240 250 260 270 280 290 300 310 320

| | | | | | | | | | **┌** | | **┐**| | |

Mpu_EpCAM TTRYQ-LDPKYITNILYEND--LITIDLMQNSSQKTQSDVDIADVAYYFEKDVKDESLFH-SN---RMDLR---INGEQLDLEPGRTAIYYVDEKPPEFSMQGLQAGIIAVIVVVSLAVIAGIVVLVISRKNRMAKYEKAEIKEMGEMHRELNA-----

Oro_EpCAM TTRYQ-LDPKYITNILYEND--LITIDLMQNSSQKTQNDVDIADVAYYFEKDVKDESLFH-SS---RMDLK---VNGEQLDLDPGRTAIYYVDEKPPEFSMQGLQAGIIAVIVVVTLAVIAGIVVLVISRKNRMAKYEKAEIKEMGEMHRELNA-----

Ame_EpCAM TTRYQ-LDPKYITNILYEND--LITIDLMQNSSQKTQNDVDIADVAYYFEKDVKDESLFH-SN---RMDLR---VNGEQLDLDPGRTAIYYVDEKPPEFSMQGLQAGIIAVIVVVTLAVIAGIVVLVISRKNRLAKYEKAEIKEMGEMHRELA------

Cfa_EpCAM KNRYQ-LDPKYITNILYEND--LITIDLMQNSSQKAQNDVDIADVAYYFEKDVKDESLFH-SS---KMDLR---VNGEQLDLDPGRTAIYYVDEKPPEFSMQGLQAGIIAVIVVVTLAVIAGIVVLVISRKNRMAKYEKAEIKEMGEMHRELNA-----

Fca_EpCAM TTRYQ-LDPKYITNILYEND--LITIDLVQNSSQKTQNDVDIADVAYYFEKDVKDESLFH-SN---KMDLR---VNGEQLDLDPGRTAIYYVDEKPPEFSMQGLQAGIIAVIVVVTLAIIAGIVVLVISRKNRMAKYEKAEIKEMGEMHRELNA-----

Bta_EpCAM TNRYQ-LDPKYITNILYEND--VITIDLVQNSSQKTQNDVDIADVAYYFEKDVKDESLFH-SK---RMDLR---VNGELLDLDPGRTSIYYVDEKPPEFSMQGLQAGIIAVIVVVVVAIIAGIIVLVVSRKKSMTKYEKAEIKEMGEMHRELNA-----

Oar_EpCAM TSRYQ-LDPKYITNILYEND--VITIDLVQNSSQKTQNDVDIADVAYYFEKDVKDESLFH-SK---RMDLK---VNGELLDLDPGQTSIYYVDEKPPEFSMQGLQAGIIAVIVVVVVAIIAGIIVLVVSRKKSMTKYEKAEIKEMGEMHRELNA-----

Oor_EpCAM TSRYQ-LDPKYITNILYEND--VIVIDLVQNSSQKTQNDVDIADVAYYFEKDVKDESLFQ-SN---RMDLR---VNGELLDLDPSRTSIYYVDEKPPEFSMQGLQAGIIAVIVVVAVAITAGIAVLVVSRKKRMAKYEKAEIKEMGEMHRELNA-----

Ttr_EpCAM TSRYQ-LDPKYITNILYEND--VIVIDLVQNSSQKTQNDVDIADVAYYFEKDVKDESLFQ-SN---KMDLR---VNGELLDLDPSRTSIYYVDEKPPEFSMQGLQAGIIAVIVVVAVAITAGIAVLVVSRKKRMAKYEKAEIKEMGEMHRELNA-----

Ssc_Epcam TDRYQ-LDPKYITNILYEND--IITIDLVQNSSQKTLNEVDIADVAYYFEKDVKDESLFH-SK---RMDLR---VNGELLDLDPGQTSIYYVDEKPPEFSMQGLQAGIIAVIAVVAIAIVAGIIVLIVSTKKRRAKYEKAEIKEMGEMHRELNA-----

Vpa_EpCAM TSRYQ-LDPKYITNILYEND--VITIDLVQNSSQKTQNDVDIADVAYYFEKDVKDESLFY-SK---RMDLK---VNGELLDLDPGRTSIYYVDEKPPEFSMQGLQAGIIAVIVVVAIAIIAGIVVLVISRKKRMAKYEKAEIKEMGEMHRELNA-----

Eca_EpCAM TNRYQ-LDPKYIADILYEND--IITIDLMQNSSQKTQNDVDIADVAYYFEKDVKDESLFH-SN---RMDLR---VNGEQLDLDPGRTSIYYVDEKPPEFSMQGLQAGIIAVIVVVIIAIIAGIVVLVISRKNRMAKYEKAEVKEMGEMHRELNA-----

Mlu_EpCAM TTRYQ-LDSKYIGNILYEND--IITIDLVQNSTQKMQNDVDIADVAYYIEKDVKDESLFH-SK---RMDLR---VDGEQLDLDPGRTAIYYVDEKPPEFSMQGLKAGIIAVIVVVAIALIAGIVVLVISTKKRKTKYEKAEIKEMGEMHRELHG-----

Laf_EpCAM TTRYQ-LDRKYITNILYEND--IITIDLMQNSSQKTQDDVDIADVAYYFEKDVKGESLFY-SG---TMDLE---VNGEQLDLDPARTSIYYVDEKGPEFSMKGLTAGIIAVIVVVIIAIIAGIAVLVISRKKRMAKYEKAEIKEMGEMHRELNA-----

Ppa_EpCAM TTRYQ-LDPKFITNILYENN--VITIDLVQNSSQKTQNDVDIADVAYYFEKDVKGESLFH-SK---KMDLT---VNGEQLDLDPGQTLIYYVDEKAPEFSMQGLKAGVIAVIVVVVIAVVAGIVVLVFSRKKRMAKYEKAEIKEMGEMHRELNA-----

Ptr_EpCAM TTRYQ-LDPKFITNILYENN--VITIDLVQNSSQKTQNDVDIADVAYYFEKDVKGESLFH-SK---KMDLT---VNGEQLDLDPGQTLIYYVDEKAPEFSMQGLKAGVIAVIVVVVIAVVAGIVVLVFSRKKRMAKYEKAEIKEMGEMHRELNA-----

Ggo_EpCAM TTRYQ-LDPKFITNILYENN--VITIDLVQNSSQKTQNDVDIADVAYYFEKDVKGESLFH-SK---KMDLT---VNGEQLDLDPGQTLIYYVDEKAPEFSMQGLKAGVIAVIVVVVIAVVAGIVVLVISRKKRMAKYEKAEIKEMGEMHRELNA-----

Hsa_EpCam TTRYQ-LDPKFITSILYENN--VITIDLVQNSSQKTQNDVDIADVAYYFEKDVKGESLFH-SK---KMDLT---VNGEQLDLDPGQTLIYYVDEKAPEFSMQGLKAGVIAVIVVVVIAVVAGIVVLVISRKKRMAKYEKAEIKEMGEMHRELNA-----

Nle_EpCAM TTRYQ-LDPKFITNILYENN--VITIDLVQNSTQKTQNDVDIADVAYYFEKDVKGESLFH-SK---KMDLT---VNGEQLDLDPGQTLIYYVDEKAPEFSMQGLKAGVIAVIVVVVIAIIAGIVVLVISRKKRMAKYEKAEIKEMGEMHRELNA-----

Mml_EpCAM KTRYQ-LDPKFITNILYEDN--VITIDLVQNSSQKTQNDVDIADVAYYFEKDVKGESLFH-SK---KMDLR---VNGEQLDLDPGQTLIYYVDEKAPEFSMQGLKAGVIAVIVVVVIAIVAGIVVLVISRKKRMAKYEKAEIKEMGEIHRELNA-----

Pan_EpCAM KTRYQ-LDPKFITNILYEDN--VITIDLVQNSSQKTQNDVDIADVAYYFEKDVKGESLFH-SK---KMDLR---VNGEQLDLDPGQTLIYYVDEKAPEFSMQGLKAGVIAVIVVVVIAVVAGIVVLVISRKKRMAKYEKAEIKEMGEIHRELNA-----

Cja_EpCAM TTRYH-LDGKFITNVVYENN--VITIDLVQNSSQKTQNDVDIADVAYYFEKDVKGESLFHPSK---KMDLR---VNGEQLDLDPGQTLIYYVDEKAPEFSMQGLKAGVIAVIVVVVIAIVAGIVVLVVSRKKRMAKYQKAEIKEMGEMNRELNA-----

Ocu_EpCAM SSRYL-LDPKFITNILYENN--VITIDLVQNSSQKTQNDVDIADVAYYFEKDVKGESLMQSSN---KMELK---VNGEQVELDPGSTLIYYVDEKAPEFSMQGLKAGIIAVIVVVALAIIAGVVVLVISRKKRMAKYEKAEIKEMGEMHREYDG-----

Mmu_EpCAM TSRYK-LNQKFIKNIMYENN--VITIDLMQNSSQKTQDDVDIADVAYYFEKDVKGESLFHSSK---SMDLR---VNGEPLDLDPGQTLIYYVDEKAPEFSMQGLTAGIIAVIVVVSLAVIAGIVVLVISTRKKSAKYEKAEIKEMGEIHRELNA-----

Rno_EpCAM ASRYM-LNPKFIKSIMYENN--VITIDLMQNSSQKTQDDVDIADVAYYFEKDVKGESLFHSSK---SMDLR---VNGELLDLDPGQTLIYYVDEKAPEFSMQGLTAGIIAVIVVVVLAVIAGIVVLVISTRKRSAKYEKAEIKEMGEIHRELNA-----

Cgr_EpCAM MSRYK-LNQKFIKNILYENN--VITIDLMQNSSQKTQDDVDIADVAYYFEKDVKGESLFHHSK---KMDLR---VNGEQLDLDPGQTLIYYVDEKAPEFSMQGLKAGIIAVIVVVVLAIIAGVVVLVMSTRKRSAKYEKAEIKEMGEIHRELNA-----

Cpo_EpCAM TKRYL-LNPKFITDIVYENN--VIIIDLMQNSSQKTQNDVDIADVAYYFEKDVKGESLFS-SK---KMDLR---VNGEPLDLDPAQTLIYYVDEKPPEFSMQGLTAGIIAVIVVVILAIIAGIVVLIISRKKKTTKYEKAEIKEMGEMHRELNA-----

Mdo_EpCAM QRRYQ-LNKKYVQNILYEND--FITIHLKQNSSQKSTNDVDIADVAYYFEKDVKGESLFF-SS---KINLD---INGEKLDLDPAQTLIYYVDEKAPEFSMQGLTAGVIAVIVVVVLALVAGIVVLIVSRKKRRTKYEKAEIKEMGEMHRELS------

Sha_EpCAM EHRYQ-LNQKYIKEITYEND--IITIELMQNGTEKRSNDVDIVDVAYYFEKDVKGESLFY-SS---KLNLD---INGEMLDLDPAQTLIYYLDEKAPEFSMQGLQAGIIAVIVVVVLALVAGIVVLVMSRKKRKTKYEKAEIKEMGEFHREPSA-----

Cad_EpCAM TDRYL-LDEKHIS-FLHEEP--LIIINLKQNVSEKGNTDVDIADVAYYFEKDIKEDS-IITG----GFGIN---VVGKPLQFEN--TVIYYVDDKAPEFVMKRLTPGLIAVIVVIVLAIVAGILVLIFTRRKS-GKYEKAEVKEMNEMHRELNS-----

Psi_EpCAM RRRYL-LEPKYIHDVVYEKP--LIIIELKQNVSQKSSQDVDIADVAYYFEKDVKGDSLLVTN----PLVIN---VSEEPLKLDQ--TLIYYVDEKPPEFSMKRLTAGVIAVIVVVVLAIVAGIIVLVITRRRR-GKYEKTEIKEMNEMQRELNP-----

Gga_EpCAM TSRYM-LDGRYISGVVYENP--TITIDLKQNSSDKTPGDVDITDVAYYFEKDVKDDSIFLNN----KLNMN---IDNEELKFDN--MMVYYVDEVPPEFSMKSLTAGVIAVIVIVVLAIVAGIIGLVLSRRRK-GKYVKAEMKEMNEMHRGLNA-----

Mga_EpCAM TSRYM-LDGRYITGVTYENP--SVTIDLKQNSSDKSSGDVDIADVAYYLEKDVKGDSIFLN-----ALNMS---IDNEALTFEN--VMVYYVDEVPPEFSMKSLTAGVVAVIVIVVLAVVAGIIGLVLSRRKK-GKYVKAEMKEMNEMHRGLNA-----

Tgu_EpCAM TRRYQ-LDGRYIASVLYENP--YITIDLKQNSSAKSIGDVDIADVAYYFEKDVKGQSIFHNNA---GLNVS---IDNEPVKFEK--TVVYYVDEIAPEFSMKSLTPGLIAVIVVIVIAVVAAIVVLVLTRRRK-GKYVKAEMKEM-EMHRGLNA-----

Aca_EpCAM QNRYQ-LRQHFIPAVKYDYP--FIQIELKQDLLQKTYRDVDIADVAYYFEKDIKRTSLFHFSN---AFNLS---VSGEPLDIEE--ILIYYIDEKPPEFSMKQLTPGIIAVVVVVVLAFITGITMFVFNRWRKTGKYEKVEIKEMGEMKREENA-----

Xla_EpCAMa VNRYG-LPEKCVSVELEGPS--LIYVDLKQNGSQKLPGEVDITDVAYYMEKDIKGDSLFHPDE---KFEIL---VNGNNFAVKEP--IIYYIDEKPHEISMKHLTPGVIAVIVVVVLAIVALIAVLIFTRRKR-GKYQKAEMKELNEMQQEAST-----

Xla_EpCAMb LNRYG-LPEKYVSVELEGSS--FIYIDLKQNGTQKLPGEVDITDVGYYMEKDIKGDPLFHPDE---KFEIL---VNGKNFGVKEP--VIYYVDEKPHEITMKHLTPGVIAVIVVVVLAVVALIAVLIFTRRKK-GKYQKAEMKELNEMQKEVST-----

Xtr_EpCAM TNRYG-LPDKYISVELETP---LIYIDLKQNTSQKLPGEVDITDVAYYMEKDVKGDSLFPANN---QFQIL---ANGNKISVKEP--MIYYIDEKPHEISMRHLTPGVIAVIVVVVLAVVALIAVLIFTRRKK-ARYQKAEMKELNEMQKEVST-----

Aam_EpCAM NSRYQ-LDKQLIDKVQYDPAARLIVVDVKKPMGDRT---IDLSRTAYYMEKDVKVLPLFRAQS---KFEPV---ADGHKLEMEK--ILVYYVDEEPPTFTMKHLTGGIIAVIVVVVLAVVAGLLAVFFVQKR------QRRYKKTQQ--REMDNM----

Asa_EpCAM NSRYQ-LDKQLIDKVQYDPAARLIVVDVKKPMGDRT---IDLSRTAYYMEKDVKVLPLFRAQS---KFEPV---ADGHKLEMEK--ILVYYVDEEPPTFTMKHLTGGIIAVIVVVVLAVVAGLLAVFFVQKR------QRRYKKTQQ--REMDNM----

Oni_EpCAMa NKRYQ-MNPSLVKEIQYDPDARMIVVDVNKVKGDRS---YDLSKMAYYMEKDVKILPLFTDQSR--RFTPS---VDGQTLEMEN--IVVYYVDEEAPTITMKHLTGGIIAVIVVVVLAVVAGLLAVFFVQKR------QRKYKKTQQ--REMEQM----

Oni_EpCAMb NKRYQ-MNPSLVKEIQYDPDARMIVVDVNKEKGDRT---QDLSTMAYYMEKDVKVLPLFTDQSR--SFTP----VDGQNLEMEK--IIVYYVDEEAPTITMKHLTGGIIAVIVVVVLAVVAGLLAVFFVQKR------QRKYKKTQQ--REMEQM----

Tru_EpCAM NKRYKNFNKDLVKEVQYDPDARMIVVDVKKEIGDRT---VDLTQMAYYMEKDVKVLPLFRNG----KFEPK---VGDQILDMEN--ILVYYVDEEAPTFTMKNLSGGIIAVIVVVVLAVVIGLVLLFFLNKRG-----KKRYNKTQQ--REMEAMS---

Tni_EpCAM NKRYQNFNKDLVDSVKYDPDARMIVVDVKKEIGDRT---ADVTQMAYYMEKDVKILPLFKSQE---KFAPV---VGGQKLEMEN--ILVYYVDEEAPTFTMQNLSGGIIAVIVVVVLAVVIGLLVLFFLRKRD-----KKRYNKTQQ--REMDAM----

Gac_EpCAM NRRYTNFDRKLVDNVEYDADSRLIVVDVKKAEGERQ---TELANMAYYMEKDVKVSPLFKNPN---KFAPV---IGGQTLEMEN--ILVYYVDEEPPTFTMKNLSAGIIAVIVVVVLAVVAGLLVLFFARKRQ-----NQKYNKAQQ--REMDPM----

Omo_EpCAM QNRYK-FDKNLVEDIKYDPEARLIVVDVKKNKGERG---TDMAGLAYYMEKDVKVLPLFKDQT---KFEPV---VNGQKLEIEN--ILVYYVDEEAPTFTMKRLTAGVIAVIVVVILAVLAGLLVLFFARRRE-----QQKYSKADH--REMDAIQTTN

Cid_EpCAM LQRYK-LDKEFVKTVEYDKDGRIIVVDVKKKMEDRV---TDLSLMAYYLEKDVKVLPLFHDDKQ--KFEVS---VAGGNVTMEN--ILVYYVDEKAPTFTMQKLTGGIIAVIVVVSLIVLAGLLVLFFIARRQ-----KAQYSKAQA--REMETMS---

Dre_EpCAM QQRYG-LDKKLVSEVQYDKDGRLIVVDVKKDENDRT---TDLSLMTYYMEKDIKVLPLFWNG-Q--PFEVD---VPGTKVSMEN--VLIYYVDDKAPTFTMQKLTGGIIAVIVVVSLIVIGGFLVLFFLARRQ-----KAQYSKAQA--REMETIS---

Ola_EpCAM QARYK-FDKSLVKEVEYDADAKKIQVDIKKDKNDTV---IKLADMAYYMEREIKKTPMFVDQN---KFAPM---VGGQKLNFTN--ILVYYVDAEPPTFKMNRLAGGVIAAIVVVLLIVAAGLLLLFFWNRR------NKKYRKTQQTEREMENK----

Xma_EpCAM QTRYQ-VDGSLVKNVQYDPDGRYIVIDIEKEKGDRT---TNLGNMAYYMEKDLKVSPLFNNQT---KVQLN---GGSQKLDLNK--IVVYYVDEERPTITMQYLTGGIIAVIVVVVLVVVLGLLALFFVNRKR-----QQRYSKTQQ--REQS------

Cmi_EpCAM VEEYQ-VAKGYIEKVEYFKDDNTIILDLKQNITVQTP--SDLATSAYYLEKDIKDSSFYTTPAGSRSIKADGIDVDGVKVTFTK--ALVYYVDERGPEFSMKQLTPGVIAVIVVVVLAIIAGLVVLVLSRKKM----KSHMYQKAEG--RELDEMQK--

.* . : : :.: : .: **:*:::* . . . . :**:* : *. * *::*.:.:: : . . ... . . : :

β

γ

ε

α
