# Supplementary material for: Regulated Intramembrane Proteolysis and Degradation of Murine Epithelial Cell Adhesion Molecule mEpCAM
Source: PLoS One. 2013 Aug 29;8(8):e71836. doi: 10.1371/journal.pone.0071836 (PMC3756971; doi:10.1371/journal.pone.0071836)
Supplement: Table S1 — Comparison of ADAM and BACE1 cleavage sites in described substrates. m: murine; h: human, aa: amino acids. (DOCX) [file pone.0071836.s002.docx]

**Table S1:** Comparison of ADAM and BACE1 cleavage sites in described substrates. m: murine; h: human, aa: amino acids.

| **ADAM cleavage sites** | | | | | | | | | | | | | | |
| --- | --- | --- | --- | --- | --- | --- | --- | --- | --- | --- | --- | --- | --- | --- |
| **Substrate** | **Species** | **Cons. (m/h)** | **Distance to**  **TMD (aa)** | | **P3** | **P2** | | **P1** | **-Cleavage sites-** | **P1‘** | | **P2‘** | **P3‘** | |
| EpCAM | m | no | 35 | | H | S | | S |  | K | | S | M | |
| App | h | yes | 12 | | H | Q | | K |  | L | | V | F | |
| AREG | h | no | 14 | | S | E | | K |  | S | | M | K | |
| AREG | m | no | 11 | | S | M | | K |  | T | | H | S | |
| TNF-α | h | no | 20 | | A | Q | | A |  | V | | R | S | |
| TNF-α | m | no | 23 | | T | L | | T |  | L | | R | S | |
| **BACE1 cleavage sites** | | | | | | | | | | | | | | |
| **Substrate** | **Species** | **Cons. (m/h)** | **Distance to**  **TMD (aa)** | **P3** | | **P2** | **P1** | | **-Cleavage sites-** | **P1‘** | **P2‘** | | **P3‘** |  |
| EpCAM | m | yes | 29 | L | | I | Y | |  | Y | V | | D |  |
| App | h | yes | 20 | V | | K | M | |  | D | A | | E |  |
| APLP1 | h | no | 29 | K | | V | N | |  | A | S | | V |  |
| APLP2 | h | yes | 28 | K | | E | M | |  | I | F | | N |  |
| CHL1 | h | yes | 20 | I | | F | Q | |  | D | V | | I |  |
| IL1R2 | h | no | 9 | L | | S | F | |  | Q | T | | L |  |
| L1 | h | no | 35 | T | | D | Y | |  | E | I | | H |  |
| NRG1 | h | yes | 10 | I | | E | F | |  | M | E | | A |  |
| NRG3 | h | yes | 10 | I | | E | F | |  | M | E | | S |  |
| PSLG-1 | h | no | 17 | S | | N | L | |  | S | V | | N |  |
| SCN1B | m | yes | 16 | I | | H | L | |  | E | V | | V |  |
| SCN2B | h | no | 15 | I | | H | L | |  | Q | V | | L |  |
| SCN2B | h | no | 12 | Q | | V | L | |  | M | E | | E |  |
| SCN2B | m | no | 14 | I | | Y | L | |  | Q | V | | L |  |
| SCN3B | m | yes | 32 | F | | E | F | |  | E | A | | H |  |
| SCN3B | m | yes | 6 | E | | D | F | |  | T | S | | V |  |
| SCN4B | m | yes | 13 | I | | F | L | |  | Q | V | | V |  |
| ST6GAL1 | m | no | 11 | L | | T | I | |  | Q | A | | K |  |
| SELPLG | h | no | 17 | S | | N | L | |  | S | V | | N |  |
