# Supplementary material for: Regulated Intramembrane Proteolysis and Degradation of Murine Epithelial Cell Adhesion Molecule mEpCAM
Source: PLoS One. 2013 Aug 29;8(8):e71836. doi: 10.1371/journal.pone.0071836 (PMC3756971; doi:10.1371/journal.pone.0071836)
Supplement: Table S2 — Vertebrate EpCAM amino acid sequence comparisons with the Homo sapiens EpCAM sequence. Latin species names (bold, full length sequences of mature EpCAM proteins available), abbreviations used, common names, accession numbers with hyperlink, amino acid identities, similar amino acid position in % are listed. (n.a. not applicable because sequences are incomplete). (DOCX) [file pone.0071836.s003.docx]

**Table S2:** Vertebrate EpCAM amino acid sequence comparisons with the *Homo sapiens* EpCAM sequence. Latin species names (bold, full length sequences of mature EpCAM proteins available), abbreviations used, common names, accession numbers with hyperlink, amino acid identities, similar amino acid position in % are listed. (n.a. not applicable because sequences are incomplete)

| **Species name** | **Abbreviated name** | **Common name** | **Accession number** | **Identical amino acid positions (%)** | **Similarity (%)** |
| --- | --- | --- | --- | --- | --- |
| **Ailuropoda melanoleuca** | Ame | Giant panda | [XP_002912486](http://www.ncbi.nlm.nih.gov/protein/301753263?report=genbank&log$=protalign&blast_rank=11&RID=NU5UBDXU01R) | 81 | 95 |
| **Amphilophus amarillo** | Aam | Cichlid fish | [AEM64779](http://www.ncbi.nlm.nih.gov/protein/343823502) | 38 | 72 |
| **Amphilophus sagittae** | Asa | Cichlid fish | [AEM64780](http://www.ncbi.nlm.nih.gov/protein/343823504) | 38 | 72 |
| **Anolis carolinensis** | Aca | Green anole | [XP_003220220](http://www.ncbi.nlm.nih.gov/protein/327270888?report=genbank&log$=protalign&blast_rank=31&RID=NU5UBDXU01R) | 53 | 81 |
| **Bos taurus** | Bta | Cow | [NP_001030367](http://www.ncbi.nlm.nih.gov/protein/78369402?report=genbank&log$=protalign&blast_rank=21&RID=NU5UBDXU01R) | 83 | 95 |
| **Callithrix jacchus** | Cja | Marmoset | [XP_002757857](http://www.ncbi.nlm.nih.gov/protein/296223915?report=genbank&log$=protalign&blast_rank=1&RID=NU5UBDXU01R) | 89 | 96 |
| Callorhinchus milii | Cmi | Elephant shark | [AFM87639](http://www.ncbi.nlm.nih.gov/protein/afm87639) | 42 | 70 |
| **Canis lupus familiaris** | Cfa | Domestic dog | [XP_538481](http://www.ncbi.nlm.nih.gov/protein/73970139?report=genbank&log$=protalign&blast_rank=12&RID=NU5UBDXU01R) | 81 | 97 |
| **Cava porcellus** | Cpo | Guinea pig | [XP_003472983](http://www.ncbi.nlm.nih.gov/protein/348574410?report=genbank&log$=protalign&blast_rank=23&RID=NU5UBDXU01R) | 77 | 91 |
| **Cricetulus griseus** | Cgr | Chinese hamster | [XP_003499591](http://www.ncbi.nlm.nih.gov/protein/354474746?report=genbank&log$=protalign&blast_rank=14&RID=NU5UBDXU01R) | 79 | 93 |
| Crotalus adamanteus | Cad | Rattlesnake | [J3S8M8](http://www.uniprot.org/uniprot/J3S8M8) | 56 | 82 |
| **Ctenopharyngodon idella** | Cid | Green carp | [ADB25057](http://www.ncbi.nlm.nih.gov/protein/ADB25057) | 41 | 72 |
| **Danio rerio** | Dre | Zebrafish | [NP_001017593](http://www.ncbi.nlm.nih.gov/protein/62955155?report=genbank&log$=protalign&blast_rank=67&RID=NU5UBDXU01R) | 37 | 73 |
| **Equus caballus** | Eca | Horse | [XP_001917830](http://www.ncbi.nlm.nih.gov/protein/194220752?report=genbank&log$=protalign&blast_rank=18&RID=NU5UBDXU01R) | 82 | 96 |
| **Felis catus** | Fca | Domestic cat | [XP_003984015](http://www.ncbi.nlm.nih.gov/protein/XP_003984015) | 84 | 97 |
| **Gallus gallus** | Gga | Chicken | [NP_001012582](http://www.ncbi.nlm.nih.gov/protein/60302776?report=genbank&log$=protalign&blast_rank=28&RID=NU5UBDXU01R) | 58 | 86 |
| **Gasterosteus aculeatus** | Gac | Three-spined stickleback | [ENSGACP00000003457](http://www.ensembl.org/Gasterosteus_aculeatus/Transcript/ProteinSummary?db=core;g=ENSGACG00000002632;r=groupVI:1052768-1056426;t=ENSGACT00000003469) | 39 | 74 |
| **Gorilla gorilla** | Ggo | Gorilla | [XP_004029249](http://www.ncbi.nlm.nih.gov/protein/426335481?report=genbank&log$=protalign&blast_rank=8&RID=NU5UBDXU01R) | 99 | 100 |
| Heterocephalus glaber | Hgl | Naked mole-rat | [EHA98520](http://www.ncbi.nlm.nih.gov/protein/eha98520) | n.a. | n.a. |
| **Homo sapiens** | Hsa | Human | [NP_002345](http://www.ncbi.nlm.nih.gov/protein/218505670?report=genbank&log$=protalign&blast_rank=10&RID=NU5UBDXU01R) | 100 | 100 |
| **Loxodonta africana** | Laf | Elephant | [XP_003417597](http://www.ncbi.nlm.nih.gov/protein/344291758?report=genbank&log$=protalign&blast_rank=16&RID=NU5UBDXU01R) | 82 | 95 |
| Macaca fascicularis | Mfa | Crab-eating macaque | [EHH49950](http://www.ncbi.nlm.nih.gov/protein/ehh49950) | n.a. | n.a. |
| **Macaca mulatta** | Mml | Rhesus macaque | [NP_001035118](http://www.ncbi.nlm.nih.gov/protein/91064856?report=genbank&log$=protalign&blast_rank=1&RID=NUVAXFCZ014) | 94 | 98 |
| **Melleagris gallopavo** | Mga | Turkey | [XP_003203766](http://www.ncbi.nlm.nih.gov/protein/326914911?report=genbank&log$=protalign&blast_rank=29&RID=NU5UBDXU01R) | 56 | 85 |
| **Monodelphis domestica** | Mdo | Opossum | [XP_001382179](http://www.ncbi.nlm.nih.gov/protein/334312203?report=genbank&log$=protalign&blast_rank=26&RID=NU5UBDXU01R) | 71 | 90 |
| **Mus musculus** | Mmu | Mouse | [NP_032558](http://www.ncbi.nlm.nih.gov/protein/NP_032558) | 82 | 94 |
| Mustela putorius furo | Mpu | Ferret | [ENSMPUP00000010950](http://www.ensembl.org/Mustela_putorius_furo/Transcript/Summary?t=ENSMPUT00000011133;r=GL896935.1:4222452-4241633;g=ENSMPUG00000011040) | 81 | 95 |
| Myotis davidii | Mda | Mouse-eared bat | [ELK26770](http://www.ncbi.nlm.nih.gov/protein/elk26770) | n.a. | n.a. |
| **Myotis lucifugus** | Mlu | Little brown bat | [ENSMLUP00000003055](http://www.ensembl.org/Myotis_lucifugus/Transcript/Sequence_Protein?db=core;g=ENSMLUG00000003363;r=GL429773:12314001-12327515;t=ENSMLUT00000003361) | 79 | 94 |
| **Nomascus leukogenys** | Nle | White-cheeked gibbon | [XP_003262404](http://www.ncbi.nlm.nih.gov/protein/XP_003262404) | 97 | 99 |
| **Orcinus orca** | Oor | Orca whale | [XP_004265027](http://www.ncbi.nlm.nih.gov/protein/465981024?report=genbank&log$=protalign&blast_rank=17&RID=NU5UBDXU01R) | 81 | 96 |
| **Oreochromis niloticus** | Oni | Nile tilapia | [ENSONIP00000003783](http://www.ensembl.org/Oreochromis_niloticus/Transcript/Sequence_Protein?db=core;g=ENSONIG00000003020;r=GL831247.1:2255636-2267256;t=ENSONIT00000003784) | 40 | 74 |
| **Oreochromis niloticus** | Oni | Nile tilapia | [ENSONIP00000003782](http://www.ensembl.org/Oreochromis_niloticus/Transcript/Sequence_Protein?db=core;g=ENSONIG00000003020;r=GL831247.1:2255636-2267256;t=ENSONIT00000003783) | 39 | 74 |
| Orinthorhynchus anatinus | Oan | Duckbill platypus | [XP_001516292](http://www.ncbi.nlm.nih.gov/protein/xp_001516292) | n.a. | n.a. |
| **Odobenus rosmarus divergens** | Oro | Pacific walrus | [XP_004403443](http://www.ncbi.nlm.nih.gov/protein/XP_004403443) | 82 | 95 |
| **Oryctolagus cuniculus** | Ocu | Rabbit | [XP_002709770](http://www.ncbi.nlm.nih.gov/protein/XP_002709770) | 82 | 97 |
| **Oryzias latipes** | Ola | Japanese medaka | [XP_004076978](http://www.ncbi.nlm.nih.gov/protein/432901987?report=genbank&log$=protalign&blast_rank=65&RID=NU5UBDXU01R) | 36 | 73 |
| **Oryzias latipes** | Ola | Japanese medaka | [XP_004084111](http://www.ncbi.nlm.nih.gov/protein/432948649?report=genbank&log$=protalign&blast_rank=68&RID=NU5UBDXU01R) | 34 | 71 |
| Osmerus mordax | Omo | Rainbow smelt | [ACO09395](http://www.ncbi.nlm.nih.gov/protein/aco09395) | 42 | 76 |
| Otolemur garnettii | Oga | Small-eared galago | [XP_003788027](http://www.ncbi.nlm.nih.gov/protein/xp_003788027) | n.a. | n.a. |
| **Ovis aries** | Oar | Sheep | [NP_001156036](http://www.ncbi.nlm.nih.gov/protein/NP_001156036) | 83 | 95 |
| **Pan paniscus** | Ppa | Pygmy chimpanzee | [XP_003822725](http://www.ncbi.nlm.nih.gov/protein/397504272?report=genbank&log$=protalign&blast_rank=9&RID=NU5UBDXU01R) | 98 | 100 |
| **Pan troglodytes** | Ptr | Chimpanzee | [XP_515458](http://www.ncbi.nlm.nih.gov/protein/114577430?report=genbank&log$=protalign&blast_rank=13&RID=NU5UBDXU01R) | 97 | 99 |
| **Papio anubis** | Pan | Olive baboon | [XP_003908676](http://www.ncbi.nlm.nih.gov/protein/XP_003908676) | 94 | 98 |
| **Pelodiscus sinensis** | Psi | Chinese soft-shell turtle | [ENSPSIP00000017678](http://www.ensembl.org/Pelodiscus_sinensis/Transcript/Sequence_Protein?db=core;g=ENSPSIG00000015680;r=JH209340.1:3270291-3280555;t=ENSPSIT00000017757) | 58 | 83 |
| Pongo abelii | Pab | Orangutan | [XP_002812099](http://www.ncbi.nlm.nih.gov/protein/XP_002812099) | n.a. | n.a. |
| Pteropus vampyrus | Pva | Large flying fox | [ENSPVAP00000004909](http://www.ensembl.org/Pteropus_vampyrus/Transcript/Sequence_Protein?db=core;g=ENSPVAG00000005187;r=GeneScaffold_3032:475356-485329;t=ENSPVAT00000005185) | n.a. | n.a. |
| **Rattus norvegicus** | Rno | Rat | [NP_612550](http://www.ncbi.nlm.nih.gov/protein/25742698?report=genbank&log$=protalign&blast_rank=19&RID=NU5UBDXU01R) | 82 | 94 |
| Saimiri boliviensis | Sbo | Squirrel monkey | [XP_003922925](http://www.ncbi.nlm.nih.gov/protein/XP_003922925) | n.a. | n.a. |
| **Sarcophilus harrisii** | Sha | Tasmanian devil | [XP_003758850](http://www.ncbi.nlm.nih.gov/protein/395509114?report=genbank&log$=protalign&blast_rank=27&RID=NU5UBDXU01R) | 69 | 89 |
| Spermophilus tricedemlineatus | Str | Thirteen-lined ground squirrel | [ENSSTOP00000007730](http://www.ensembl.org/Ictidomys_tridecemlineatus/Transcript/ProteinSummary?g=ENSSTOG00000008631;r=JH393318.1:5019918-5032414;t=ENSSTOT00000008625) | n.a. | n.a. |
| **Sus scofa** | Ssc | Domestic pig | [NP_999584](http://www.ncbi.nlm.nih.gov/protein/47523892?report=genbank&log$=protalign&blast_rank=22&RID=NU5UBDXU01R) | 82 | 94 |
| **Taeniopygia guttata** | Tgu | Zebra finch | [XP_002195947](http://www.ncbi.nlm.nih.gov/protein/449496019?report=genbank&log$=protalign&blast_rank=30&RID=NU5UBDXU01R) | 58 | 84 |
| **Takifugus rubripes** | Tru | Japanese puffer fish | [XP_003963665](http://www.ncbi.nlm.nih.gov/protein/410900362?report=genbank&log$=protalign&blast_rank=69&RID=NU5UBDXU01R) | 39 | 74 |
| **Tetraodon nigroviridis** | Tni | Spotted green puffer fish | [ENSTNIP00000020234](http://www.ensembl.org/Tetraodon_nigroviridis/Transcript/Sequence_Protein?db=core;g=ENSTNIG00000017100;r=Un_random:95317758-95319960;t=ENSTNIT00000020465) | 40 | 73 |
| **Tursiops truncatus** | Ttr | Bottle-nosed dolphin | [XP_004313191](http://www.ncbi.nlm.nih.gov/protein/470603877?report=genbank&log$=protalign&blast_rank=15&RID=NU5UBDXU01R) | 81 | 96 |
| **Vicugna pacos** | Vpa | Alpaca | [ENSVPAP00000003209](http://www.ensembl.org/Vicugna_pacos/Transcript/Sequence_Protein?db=core;g=ENSVPAG00000003467;r=scaffold_2079:121551-132271;t=ENSVPAT00000003465) | 84 | 96 |
| **Xenopus laevis** | Xla | African clawed frog | [NP_001086975](http://www.ncbi.nlm.nih.gov/protein/148228392?report=genbank&log$=protalign&blast_rank=58&RID=NU5UBDXU01R) | 48 | 80 |
| **Xenopus laevis** | Xla | African clawed frog | [NP_001079573](http://www.ncbi.nlm.nih.gov/protein/148227480?report=genbank&log$=protalign&blast_rank=60&RID=NU5UBDXU01R) | 48 | 80 |
| **Xenopus tropicalis** | Xtr | Western clawed frog | [NP_001011149](http://www.ncbi.nlm.nih.gov/protein/58332000?report=genbank&log$=protalign&blast_rank=57&RID=NU5UBDXU01R) | 49 | 80 |
| **Xiphophorus maculatus** | Xma | Southern platyfish | [ENSXMAP00000007490](http://www.ensembl.org/Xiphophorus_maculatus/Transcript/Sequence_Protein?db=core;g=ENSXMAG00000007455;r=JH556663.1:4423029-4425800;t=ENSXMAT00000007498) | 37 | 73 |

8
